# Impact of the COVID-19 Pandemic on the Socioeconomic Inequalities in Mortality in Spanish Provinces

**DOI:** 10.1007/s44197-023-00125-0

**Published:** 2023-06-09

**Authors:** Maria A. Barceló, Marc Saez

**Affiliations:** 1 Research Group on Statistics, Econometrics and Health (GRECS), University of Girona, Carrer de la Universitat de Girona 10, Campus de Montilivi, Girona, Spain; 2 Centro de Investigación Biomédica en Red de Epidemiología y Salud Pública, Instituto de Salud Carlos III, Madrid, Spain

**Keywords:** COVID-19, Mortality, Socioeconomic inequalities, Gini index

## Abstract

**Background:**

Although many studies have assessed the socioeconomic inequalities caused by COVID-19 in several health outcomes, there are numerous issues that have been poorly addressed. For instance, have socioeconomic inequalities in mortality from COVID-19 increased? What impact has the pandemic had on inequalities in specific causes of mortality other than COVID-19? Are the inequalities in COVID-19 mortality different from other causes? In this paper we have attempted to answer these questions for the case of Spain.

**Methods:**

We used a mixed longitudinal ecological design in which we observed mortality from 2005 to 2020 in the 54 provinces into which Spain is divided. We considered mortality from all causes, not excluding, and excluding mortality from COVID-19; and cause-specific mortality. We were interested in analysing the trend of the outcome variables according to inequality, controlling for both observed and unobserved confounders.

**Results:**

Our main finding was that the increased risk of dying in 2020 was greater in the Spanish provinces with greater inequality. In addition, we have found that: (i) the pandemic has exacerbated socioeconomic inequalities in mortality, (ii) COVID-19 has led to gender differences in the variations in risk of dying (higher in the case of women) and (iii) only in cardiovascular diseases and Alzheimer did the increased risk of dying differ between the most and least unequal provinces. The increase in the risk of dying was different by gender (greater in women) for cardiovascular diseases and cancer.

**Conclusion:**

Our results can be used to help health authorities know where and in which population groups future pandemics will have the greatest impact and, therefore, be able to take appropriate measures to prevent such effects.

**Supplementary Information:**

The online version contains supplementary material available at 10.1007/s44197-023-00125-0.

## Background

Many studies have assessed socioeconomic inequalities in several health outcomes from COVID-19, concluding that low socioeconomic status, both at the individual and ecological level, is a risk factor for mortality and other health outcomes from COVID-19.

However, there are a number of issues that have barely been addressed or not addressed at all. First, have these inequalities continued to be the same, or have they decreased or increased? Second, focusing on mortality due to COVID-19, what has the impact of the pandemic been on inequalities in overall mortality and in specific causes mortality other than COVID-19? In relation to this, have the inequalities in COVID-19 mortality been greater than, less than, or equal to the inequalities in mortality from other causes?

We hypothesize that although socioeconomic inequalities in mortality from COVID-19 have existed, they may have varied over time and may not have coincided with the inequalities in mortality from other causes.

Therefore, in this paper we intend to assess the impact of the COVID-19 pandemic on inequalities in overall mortality and mortality from specific causes, as well as to find out if these inequalities have increased because of the pandemic. In addition, we intend to evaluate our objectives according to gender and at a geographical level smaller than a country level, specifically at the level of Spanish provinces.

To exemplify our hypotheses and objectives, we carried out a bibliographic search in PubMed in early July 2022 using the keywords ‘inequalities’, ‘mortality’ and ‘COVID-19’ and found 558 studies spanning from very early in the pandemic (April 2020) until (the end of June 2022. Among these studies, we found four systematic reviews [1–4] and two non-systematic reviews [5, 6], which included studies at both the ecological and individual levels and of almost all research designs (both prospective and retrospective cohorts, case–control, cross-sectional and longitudinal studies), published between December 2019 and June 2022. At the beginning of the pandemic, publications such as those from the British Office for National Statistics (ONS), had already shown that the most economically deprived areas had higher COVID-19 mortality rates [7]. In the same vein, the Nuffield Trust, again very early on in the pandemic, produced a graph showing that COVID mortality in the most deprived 10% of areas was double that in the least deprived 10% of areas [8].

That said, very few studies (and none of the systematic reviews) assess whether these inequalities continued to be the same, have decreased or increased. In fact, studies evaluating excess mortality during the COVID-19 pandemic [9–19] would have to be used to find some kind of answer even though only a few of them directly address this issue [9, 11, 13, 17, 19]^.^

While very few studies have addressed the impact of the pandemic on inequalities in overall mortality and in specific causes mortality other than COVID-19, most of them have, in fact, done it indirectly [9, 11]. The Nuffield trust graph showed that, as in the case of the rates of deaths involving COVID-19, the most deprived areas of England compared with the most affluent in deaths have twice the rate of deaths from suicide at all ages, of conditions such as liver disease and cancer for people aged under 75 (all corresponding to the period 2015–2017), as well as overall mortality rates in all ages (corresponding to 2018) [8].

Lastly, very few studies assess inequalities in mortality in a geographic area smaller than a country [9–11, 11, 13, 19–30].

## Methods

### Study Area and Study Period

We used a mixed longitudinal ecological design in which we observed mortality in the 54 provinces into which Spain is divided, from 2005 to 2020.

### Outcome Variables

We considered mortality from all causes (ICD-10: 00A, 00B, 00C, 001-102), not excluding and excluding mortality from COVID-19 (ICD-10: 00A, 00B, 00C), and cause-specific mortality. The causes that we contemplated were, in decreasing order based on the number of deaths: cardiovascular diseases (ICD-10: 053-061), cancer (ICD-10: 009-041), respiratory diseases (excluding influenza and COVID-19) (ICD-10: 063-067), Alzheimer’s (ICD-10: 051), digestive system diseases (ICD-10: 068-072), mortality due to mental and behavioural disorders (excluding suicides) (ICD-10: 046-049), infectious diseases (excluding influenza and COVID-19) (ICD-10: 001-008), diabetes mellitus (ICD-10: 044), external causes (excluding suicides) (ICD-10: 090-097, 099-102), diseases of the urinary system (ICD-10: 077-080), suicides (ICD-10: 098), and influenza (ICD-10: 062). We also considered mortality from causes other than those indicated above.

Data were obtained from death statistics according to cause of death from the Spanish National Institute of Statistics (INE) [31].

The INE follows Article 274 of the Regulations of the Spanish Civil Registry Law which states that, ‘the doctor who has assisted the deceased in their last illness or any other who recognizes the corpse will immediately send to the Registry a death certificate in which, in addition to the name, surnames (…), will state that there are unequivocal signs of death, its cause and, with the precision required by the protocol, register the date, time and place of death’. In addition, as established in Article 20 of the aforementioned Regulations, the Civil Registrar Managers, through their Provincial Delegations, send the National Institute of Statistics the bulletins on births, marriages, deaths or other registrable events [32].

The units of measurement of the outcomes are the number of deaths between 2005 and 2020 in each of the Spanish provinces.

### Socioeconomic and Demographic Variables

We considered socioeconomic and demographic variables as variables that could influence the evolution of the risk of dying. The socioeconomic variables were the average income per person (in Euros) and the Gini index (in percentage). Both were built as the average of the years 2015, 2016, 2017 and 2018 (Source: INE [33]). The demographic variable was the percentage of population aged 65 and over in 2020 (Source: INE [34]). In all cases, the unit of analysis was the province.

Average net income per person and the Gini index were categorized first into quartiles, and then these were grouped into first and second (reference category for the Gini index), and third and fourth (reference category for average net income per person).

### Data Analysis

We were interested in analysing the trend of the outcome variables according to inequality. For this purpose, we specified generalized linear mixed models (GLMM) with variable response with two links from the Poisson family: negative binomial and zero inflated Poisson. Both allowed for the presence of heteroskedasticity in the response variable (i.e., non-constant variance across provinces and/or over time) and, in the latter case, an excess of zeros in mortality for some provinces and/or years for some causes of mortality (i.e., no deaths).

For each outcome variable we chose the link in which the Bayesian model selection method of Watanabe-Akaike information criterion (WAIC) [35] of the fitted model was lower.

In the GLMM, we assessed the trend in mortality (for all causes and for the specific causes indicated above) distinguishing between the most and least unequal provinces, approximating inequality with the Gini index.

As variables that could influence the risk, we included the average income per person of the provinces and we controlled for both observed and unobserved confounders. As observed confounders we considered the percentage of population aged 65 and over and the population of the province in each of the years under study.

In detail, conditional to the true risk in the province $i$ on year $t$, the cases of the response variable (${Y}_{it}$) occurring in each of the provinces in each year was distributed as a negative binomial or as a zero inflated Poisson.

$${Y}_{it}\left|{\theta }_{it}\right.\sim Negative\, binomial\left({\theta }_{it}{Population}_{it}\right)$$

$${Y}_{it}\left|{\theta }_{it}\right.\sim Zero\, inflated \,Poisson\left({\theta }_{it}{Population}_{it}\right)$$

where ${\theta }_{it}$ denoted $E\left({Y}_{it}\right)={\theta }_{it}$; $i=1,\dots , 54$; $t$=2005, 2006,…, 2020; and ${Population}_{it}$ was the population at risk of being a case (death) in the province $i$ and on year $t$.

The link functions of the GLMMs were as follows:

$$\mathrm{log}\left({\theta }_{it}\right)={\beta }_{0}+{\beta }_{1} {income\_Q12}_{i}+{\beta }_{2} {Gini\_Q34}_{i}+\sum_{k=2}^{4}{\beta }_{3k} {Perc\_pop\_65\_or\_moreQ}_{ik} + {\eta }_{i}+{\tau }_{t }{Gini\_Q34}_{t} +offset(\mathrm{log}\left({Population}_{it}\right))$$

where the subindexes $i$ and $t$ indicated the province, and the year, respectively; *income_Q12*_*i*_ denoted whether the province is located in one of the first two quartiles of the average income per person; *Gini_Q34*_*i*_ denoted whether the province is located in one of the last two quartiles of the Gini index*; Perc_pop_65_or_moreQ*_*ik*_ the percentage of population aged 65 and over in 2020 (in quartiles, taking the first quartile as the reference category): ${\eta }_{i}, {\tau }_{t}$ denoted random effects; and $\beta s$ were the coefficients of the explanatory and control variables (${e}^{\beta }$ was the relative risk associated with each of them).

We included two random effects in the models. First, ${\eta }_{i}$, a random effect indexed on the province. This random effect was unstructured (independent and identically distributed random effects, iid), and captured individual heterogeneity, that is to say, unobserved confounders specific to the province and invariant in time.

Second, we included ${\tau }_{t}$, a structured random effect (random walk of order one, rw1) indexed on time. i.e., the evolution of the risk of dying over time. It should be noted that we allowed for this evolution to be non-linear. Following the integrated nested Laplace approximation (INLA) approach [36, 37] when, as in our case, the random effects are indexed on a continuous variable, they can be used as smoothers to model non-linear dependency on covariates in the linear predictor. With this random effect we captured the temporal dependency, that is, the trend, which we allowed to be non-linear.

Note that we included in the models this random effect interacting with Gini_Q34. In fact, for each cause we were interested in evaluating the mortality trend (possibly non-linear) distinguishing between the provinces located in the last two quartiles of the index of Gini (those with the greatest inequality) and in the first two quartiles (those with the least inequality) of the index.

Following the INLA approach, random effects were defined using a multivariate Gaussian distribution with a zero mean and precision matrix kΣ, where *k* was a constant and Σ was a matrix that defined the dependence structure of the random effects [36, 37]. In unstructured random effects (iid) Σ was a diagonal matrix of 1 s, and in random walk random effects Σ was defined assuming that increments (in rw1, $\Delta {u}_{i}={u}_{t}-{u}_{t-1}$) followed a Gaussian distribution with zero mean and a constant precision *k* [38, 39].

### Inference

Inferences were made following a Bayesian perspective, using the INLA approach [36, 37] under its experimental mode [40]. We used priors that penalize complexity (called PC priors). These priors are robust in the sense that they do not have an impact on the results and, in addition, they have an epidemiological interpretation [41].

All analyses were carried out using the free software R (version 4.2.0) [42], available through the INLA package [36, 37, 39].

All analyses were unstratified and stratified by gender.

## Results

### Baseline Characteristics

In Table 1 we show the mortality rates from all causes for the year 2018, standardized by sex and age (per 100,000 inhabitants) without stratifying and stratifying by gender. Note that (from greater to lesser difference): Melilla (only one province), the provinces of Andalusia *– Andalucía-*, Ceuta (only one province); Murcia (only one province), the provinces of Extremadura; the provinces of the Canary Islands *– Islas Canarias-*; and the provinces of the Valencian Community *– Comunidad Valenciana-*; were the ones with standardized rates higher than those of Spain as a whole. The rest (11 out of 17 autonomous communities) had lower standardized rates than in Spain. When stratified by gender, Cantabria, and Galicia (in men), and the Balearic Islands *– Illes Balears-* (in women) started to have higher standardized rates than Spain as a whole. Standardized mortality rates by cause of death (large groups), by autonomous communities and cities, and stratified by sex, are shown in Table S1 in supplementary material.

**Table 1:** Mortality rates from all causes, standardized by sex and age (per 100,000 inhabitants)

	All		Men		Women	
	Rate	Coefficient of variation	Rate	Coefficient of variation	Rate	Coefficient of variation
All Spain	832.16	0.13	1068.26	0.18	652.63	0.19
Autonomous communities						
Andalucía	953.64	0.30	1186.44	0.42	768.14	0.44
Aragón	804.45	0.75	1041.95	0.99	619.21	1.12
Asturias, Principado de	867.11	0.77	1145.36	1.02	669.74	1.13
Illes Balears	818.92	0.92	1019.47	1.26	656.70	1.35
Canarias	884.18	0.66	1081.54	0.90	717.39	0.97
Cantabria	817.88	1.13	1107.08	1.46	612.19	1.69
Castilla y León	757.05	0.54	971.46	0.71	589.29	0.81
Castilla-La Mancha	816.53	0.63	1020.37	0.84	648.37	0.92
Cataluña	807.95	0.33	1050.46	0.44	628.20	0.49
Comunitat Valenciana	879.67	0.39	1095.91	0.53	706.98	0.57
Extremadura	890.05	0.81	1127.29	1.08	694.37	1.18
Galicia	820.31	0.50	1072.22	0.66	630.73	0.73
Madrid, Comunidad de	689.04	0.40	903.99	0.55	541.98	0.58
Murcia, Región de	890.29	0.77	1112.65	1.05	714.62	1.12
Navarra, Comunidad Foral de	761.79	1.14	1012.54	1.46	582.24	1.71
País Vasco	779.49	0.59	1043.14	0.77	597.10	0.87
La Rioja	796.98	1.57	1061.47	2.00	598.52	2.40
Autonomous cities						
Ceuta	952.60	3.72	1133.55	5.23	819.45	5.31
Melilla	1,009.15	3.72	1149.75	5.39	889.35	5.20

Spain, autonomous communities and cities. Without stratifying and stratifying by gender, 2018

Provinces in each Autonomous Community

Andalucía: Almería, Cádiz, Córdoba, Granada, Huelva, Jaén, Málaga, Sevilla

Aragón: Huesca, Teruel Zaragoza

Asturias: Asturias

Illes Balears: Illes Balears

Canarias: Las Palmas, Santa Cruz de Tenerife

Cantabria: Cantabria

Castilla y León: Ávila, Burgos, León, Palencia, Salamanca, Segovia, Soria, Valladolid, Zamora

Castilla-La Mancha: Albacete, Ciudad Real, Cuenca, Guadalajara, Toledo

Cataluña: Barcelona, Girona, Lleida, Tarragona

Comunitat Valenciana: Alacant/Alicante, Castelló/Castellón, València/Valencia

Extremadura: Badajoz, Cáceres

Galicia: A Coruña, Lugo, Orense, Pontevedra

Madrid: Madrid

Murcia: Murcia

Navarra: Navarra

País Vasco: Araba/Álava, Bizcaia, Guipuzkoa

La Rioja: La Rioja

Source: INE, https://www.ine.es/jaxi/Tabla.htm?tpx=33383&L=0

**Fig. 1:**
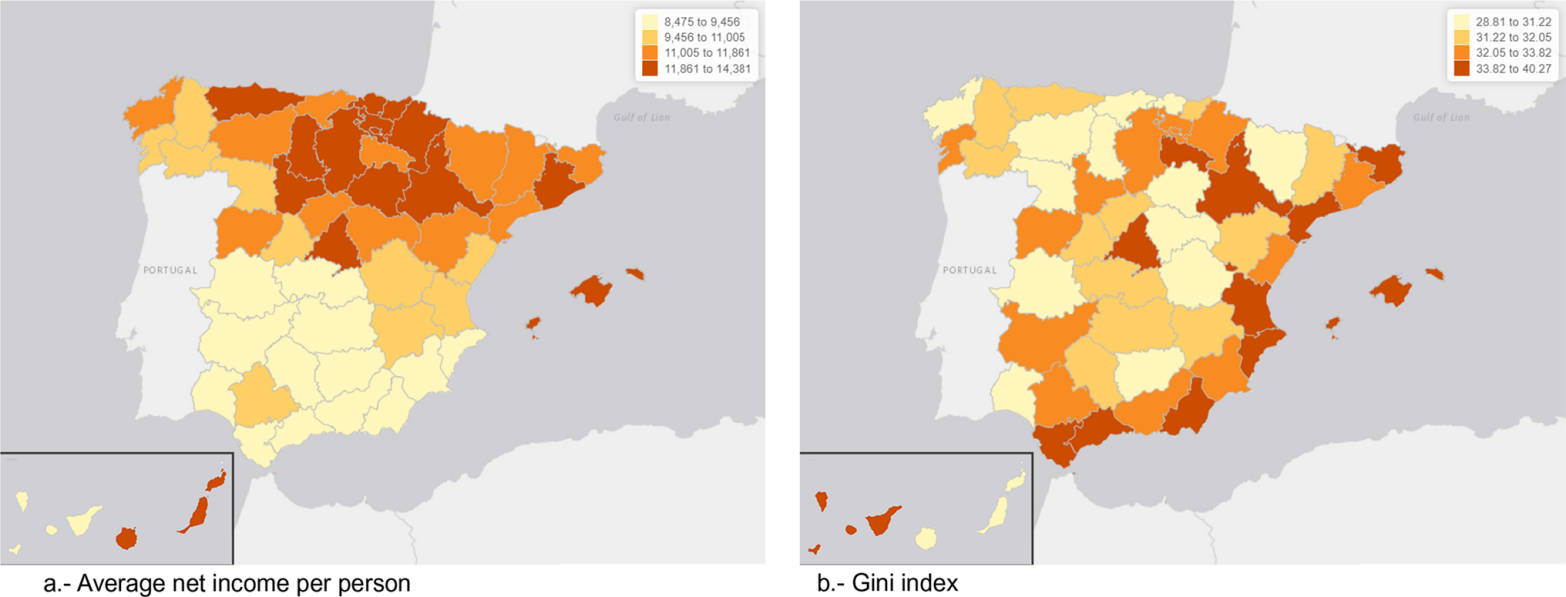
Distribution of the average net income per person and the Gini index in the Spanish provinces in 2019. Average net income quartiles (**a**) and Gini index quartiles (**b**)

In Table 2 we show a descriptive of the average net income per person and the Gini index by Spanish provinces (average 2015 to 2018) and, in Fig. 1, their distribution on a map of Spain by provinces. In the 2015–2018 period, the five provinces with the highest average net income per person were, in descending order, Madrid; all the provinces of the Basque Country (Guipuzkoa, Bizcaia and Araba); and Barcelona (Catalonia); while the five provinces with the lowest average net income, from lowest to highest income, Almería; Huelva (both in Andalusia); Badajoz (in Extremadura); Jaén; and Cádiz (both in Andalusia) (Table 2). Note that, while in Madrid, the average net income per person was 31.42% higher than the average for all of Spain; in Almería it was 22.55% lower. Net income in Madrid was 70% higher than in Almería. Regarding the Gini index, also in the 2015–2018 period, the five provinces with the highest index were, in descending order, Melilla; Madrid; Malaga (Andalusia); Balearic Islands; and La Riona; while the five provinces with the lowest index, in this case in ascending order, Soria (Castilla y León); Badajoz (Extremadura); Huesca (Aragon); Jaen (Andalusia); and Palencia (Castilla y León). Note that, while in Madrid, the index was 13.1% higher than the average for all of Spain; in Soria it was 11.82% lower. The Gini index in Madrid was 29% higher than in Soria.

**Table 2:** Descriptive of the average net income per person and the Gini index

Spain	Average net income per person (Euros)			Gini index (%)	
	10,942.70			32.67	
Autonomous community	Provinces	Average net income per person		Gini index	
		Euros	Rank (descending order)	%	Rank (descending order)
Andalucía	Almería	8474.70	52	35.08	46
Andalucía	Cádiz	8914.71	48	33.85	39
Andalucía	Córdoba	9108.63	47	31.48	20
Andalucía	Granada	9301.55	44	33.72	38
Andalucía	Huelva	8831.89	51	30.75	8
Andalucía	Jaén	8878.85	49	30.45	4
Andalucía	Málaga	9150.63	45	36.30	50
Andalucía	Sevilla	9483.78	39	32.73	32
Aragón	Huesca	11,820.91	14	30.39	3
Aragón	Teruel	11,286.12	23	31.33	16
Aragón	Zaragoza	12,397.63	8	34.71	43
Asturias. Principado de	Asturias	12,376.30	9	31.77	24
Illes Balears	Illes Balears	11,969.09	12	35.23	49
Canarias	Las Palmas	10,120.45	35	33.24	36
Canarias	Santa Cruz de Tenerife	9551.77	37	35.00	45
Cantabria	Cantabria	11,738.92	16	31.22	13
Castilla y León	Ávila	10,185.74	34	31.96	25
Castilla y León	Burgos	12,607.11	7	32.70	31
Castilla y León	León	11,582.65	18	30.69	7
Castilla y León	Palencia	11,874.68	13	30.57	5
Castilla y León	Salamanca	11,089.08	25	32.48	29
Castilla y León	Segovia	11,224.65	24	31.32	15
Castilla y León	Soria	12,177.86	11	28.81	1
Castilla y León	Valladolid	12,228.11	10	32.13	26
Castilla y León	Zamora	10,263.03	33	30.59	6
Castilla—La Mancha	Albacete	9690.35	36	31.52	22
Castilla—La Mancha	Ciudad Real	9328.81	43	31.25	14
Castilla—La Mancha	Cuenca	9506.84	38	31.14	12
Castilla—La Mancha	Guadalajara	11,449.77	21	30.84	11
Castilla—La Mancha	Toledo	9350.63	42	31.49	21
Cataluña	Barcelona	13,482.90	5	33.16	34
Cataluña	Girona	11,680.95	17	34.35	40
Cataluña	Lleida	11,483.83	20	31.55	23
Cataluña	Tarragona	11,360.60	22	34.56	42
Comunitat Valenciana	Alicante/Alacant	9130.09	46	35.12	47
Comunitat Valenciana	Castellón/Castelló	10,758.94	28	33.53	37
Comunitat Valenciana	Valencia/València	10,920.79	26	34.98	44
Extremadura	Badajoz	8860.96	50	32.47	28
Extremadura	Cáceres	9446.59	40	30.34	2
Galicia	Coruña. A	11,579.99	19	30.77	9
Galicia	Lugo	10,746.13	29	31.38	17
Galicia	Ourense	10,622.28	30	31.42	18
Galicia	Pontevedra	10,607.49	31	32.34	27
Madrid, Comunidad de	Madrid	14,380.55	1	36.95	51
Murcia, Región de	Murcia	9374.67	41	33.24	35
Navarra, Comunidad Foral de	Navarra	12,872.22	6	33.14	33
País Vasco	Araba/Álava	14,189.49	4	32.55	30
País Vasco	Bizkaia	14,236.13	3	30.83	10
País Vasco	Gipuzkoa	14,320.55	2	31.44	19
La Rioja	La Rioja	11,754.75	15	35.22	48

Autonomous cities	Provinces	Average net income per person		Gini index	
		Euros	Rank	%	Rank
Ceuta	Ceuta	10,901.75	27	34.50	41
Melilla	Melilla	10,344.00	32	40.28	52

Source: INE. https://www.ine.es/en/experimental/atlas/exp_atlas_tab_en.htm

Average 2015 to 2018

In Fig. 1 we can see that, while the distribution of the average net income per person presents a clear geographical pattern, with the first two quartiles to the north of an imaginary line that passes through Madrid in central Spain (with the exception of Galicia, in the west), the geographical distribution of the Gini index is not nearly so clear cut. For this reason, in Fig. S1 in the supplementary material, we represent a scatter graph of the Gini index against the average net income per person for the Spanish provinces. At the provincial level, a very slight downward trend can be seen, i.e., the higher the income, the lower the inequality.

### Trends in All Causes of Death

In Figs. 2a, 3, 4, 5, 6, 7, 8, 9, 10, 11, 12, 13, 14 and 15a we show the evolution of the (observed) crude mortality rate from all causes and from specific causes from 2005 to 2020. The results of the estimation of the GLMMs are shown in Tables 3, 4, 5, 6, 7 and 8 and in Figs. 2b, 3, 4, 5, 6, 7, 8, 9, 10, 11, 12, 13, 14 and 15b.

**Fig. 2:**
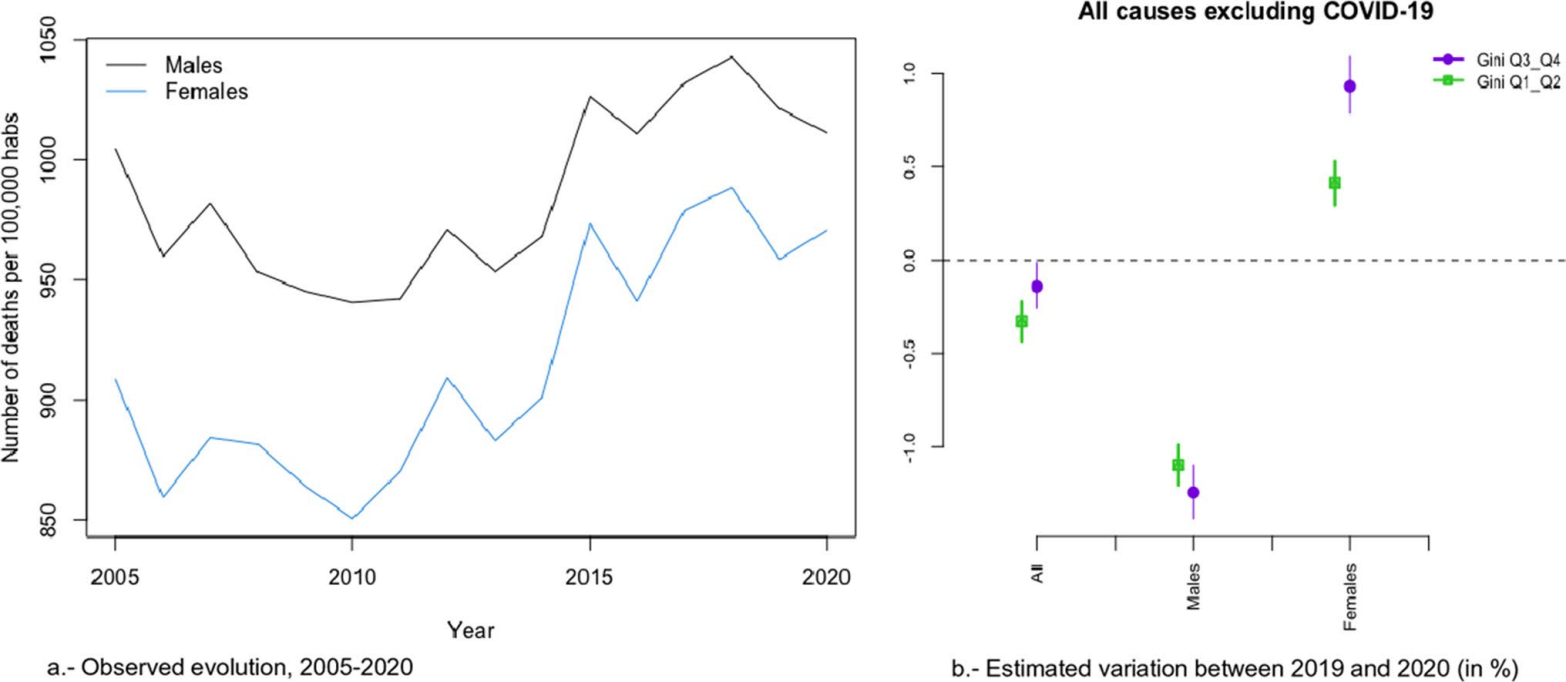
Evolution of mortality from all causes excluding COVID-19

**Fig. 3:**
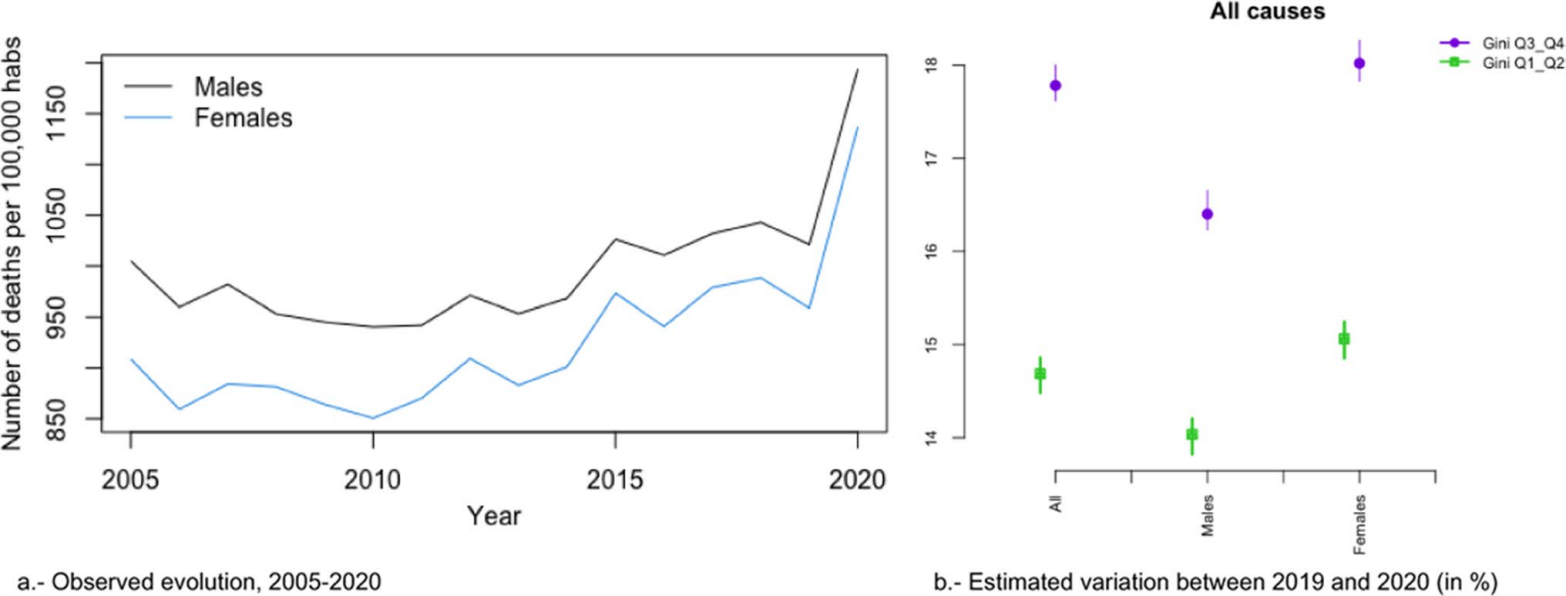
Evolution of mortality from all causes

**Fig. 4:**
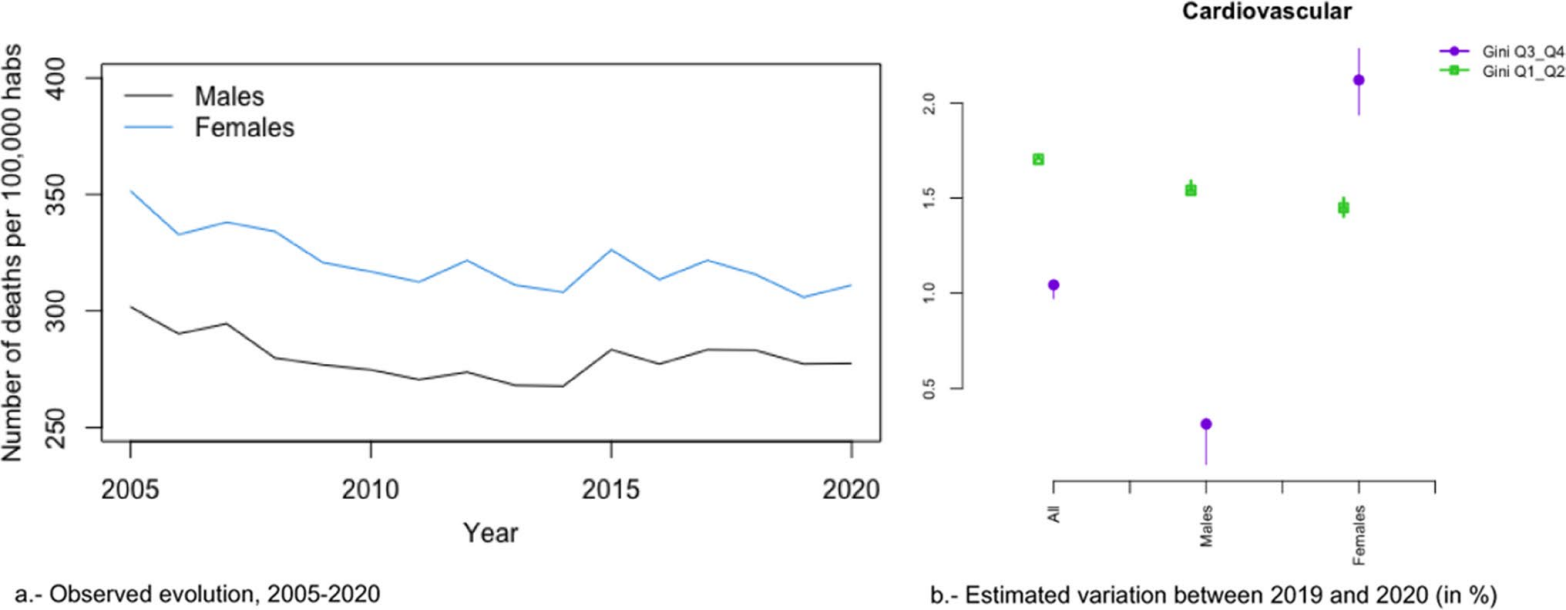
Evolution of mortality from cardiovascular diseases

**Fig. 5:**
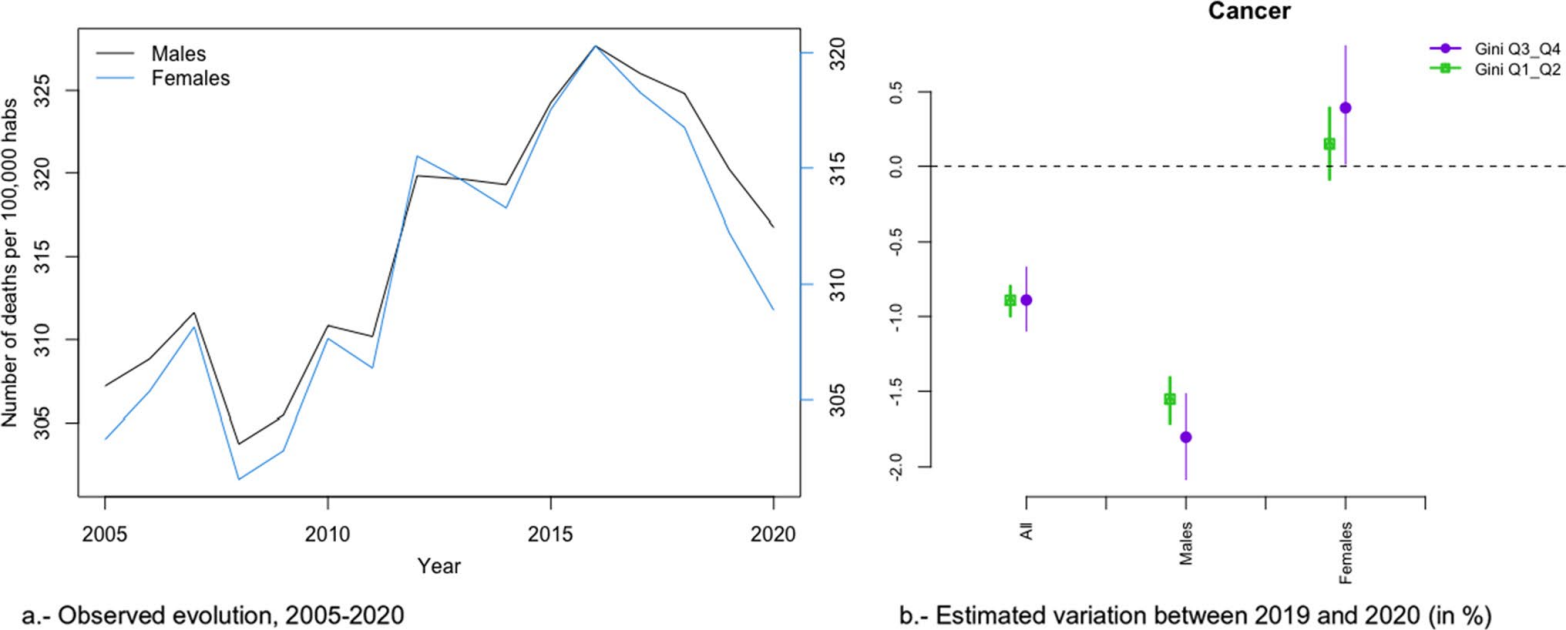
Evolution of mortality from cancer

**Fig. 6:**
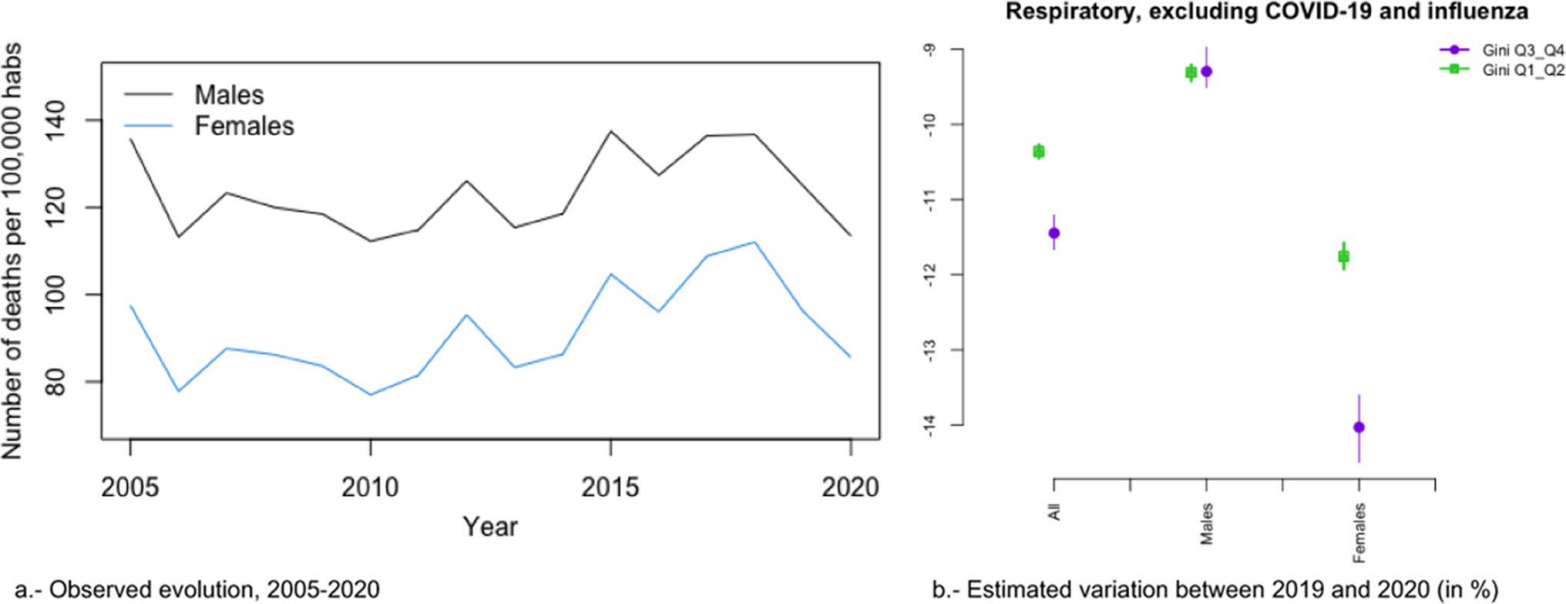
Evolution of mortality from respiratory diseases, excluding COVID-19 and influenza

**Fig. 7:**
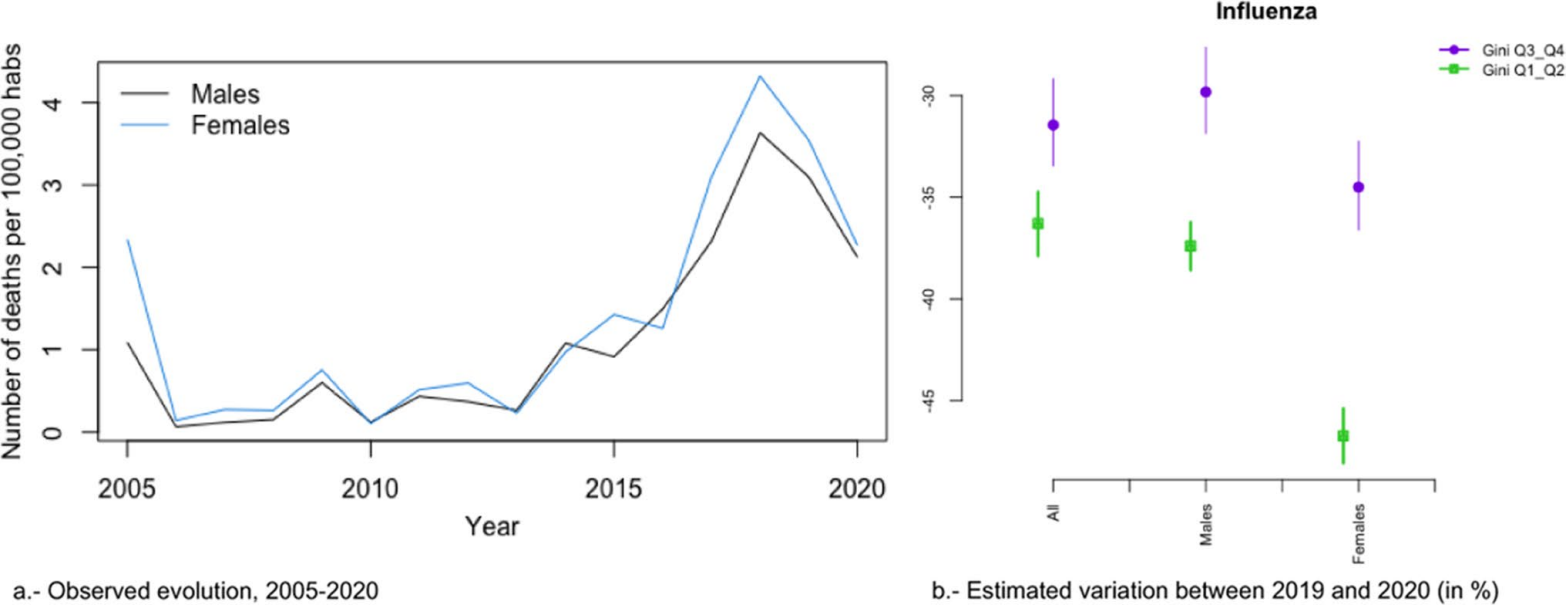
Evolution of mortality from influenza

**Fig. 8:**
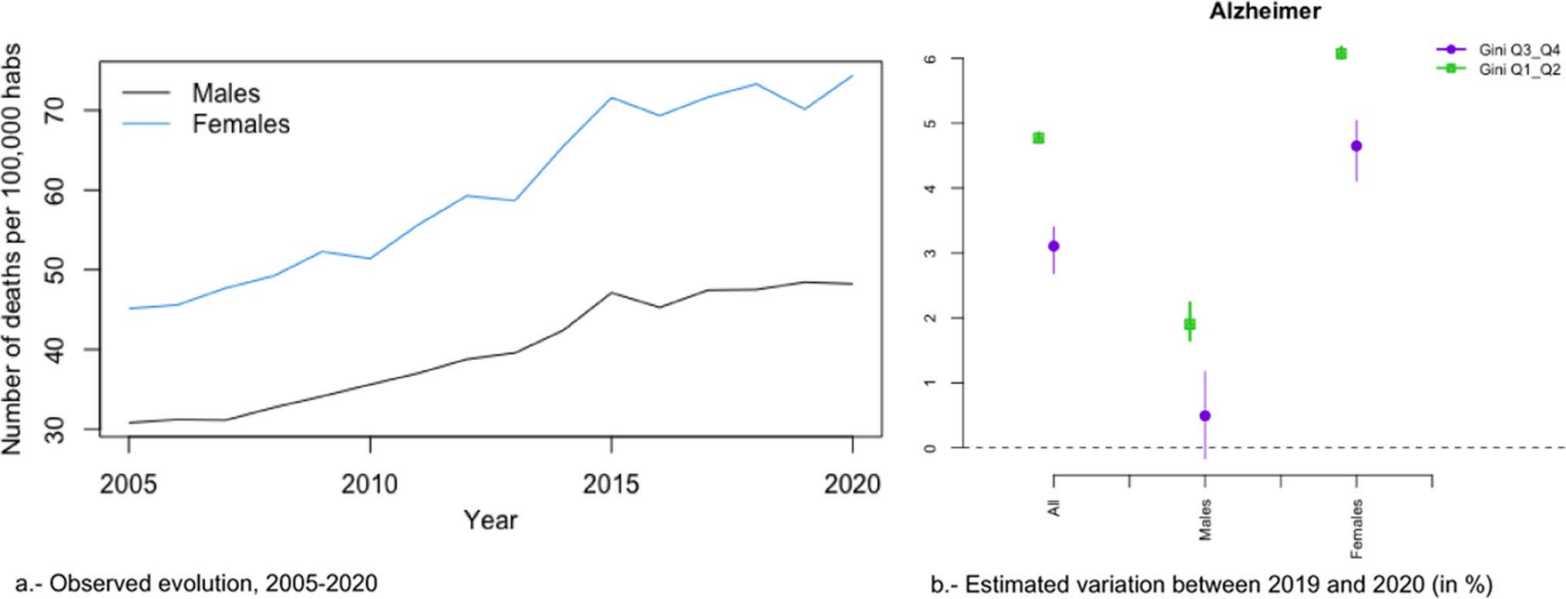
Evolution of mortality from Alzheimer’s

**Fig. 9:**
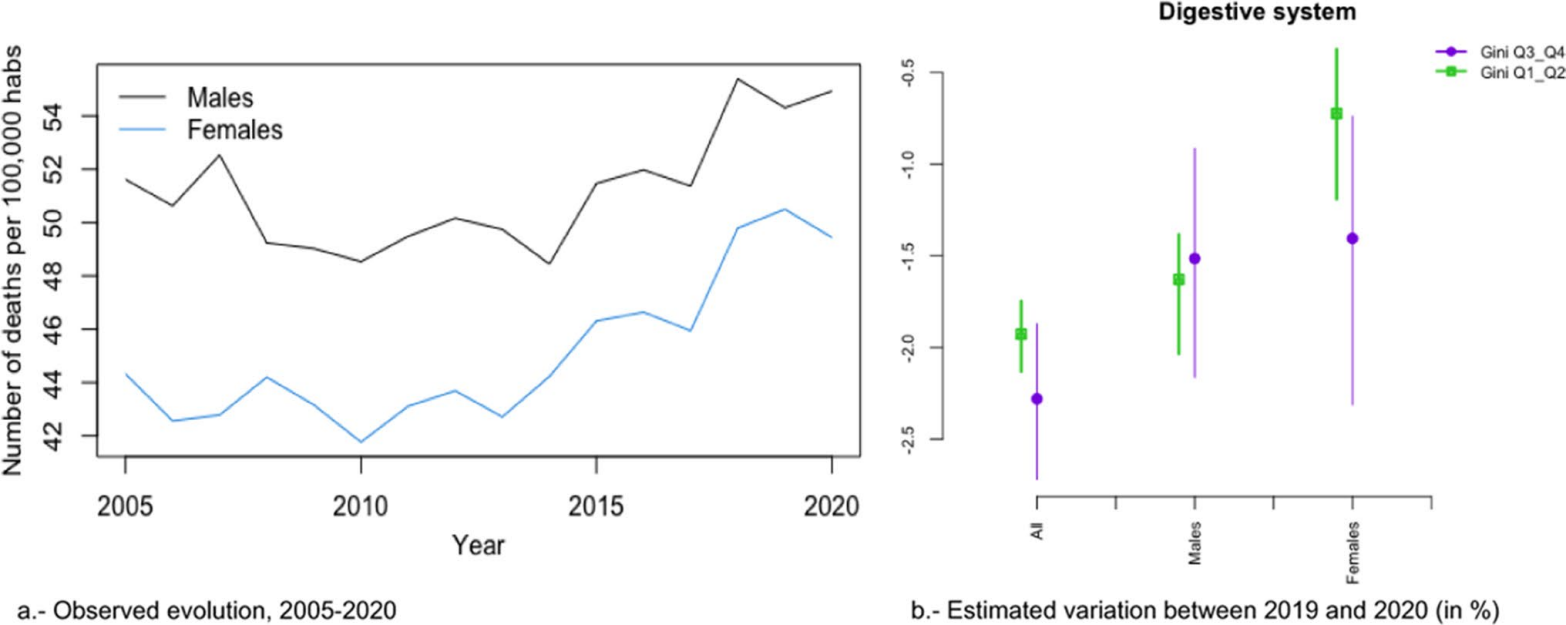
Evolution of mortality from digestive system diseases

**Fig. 10:**
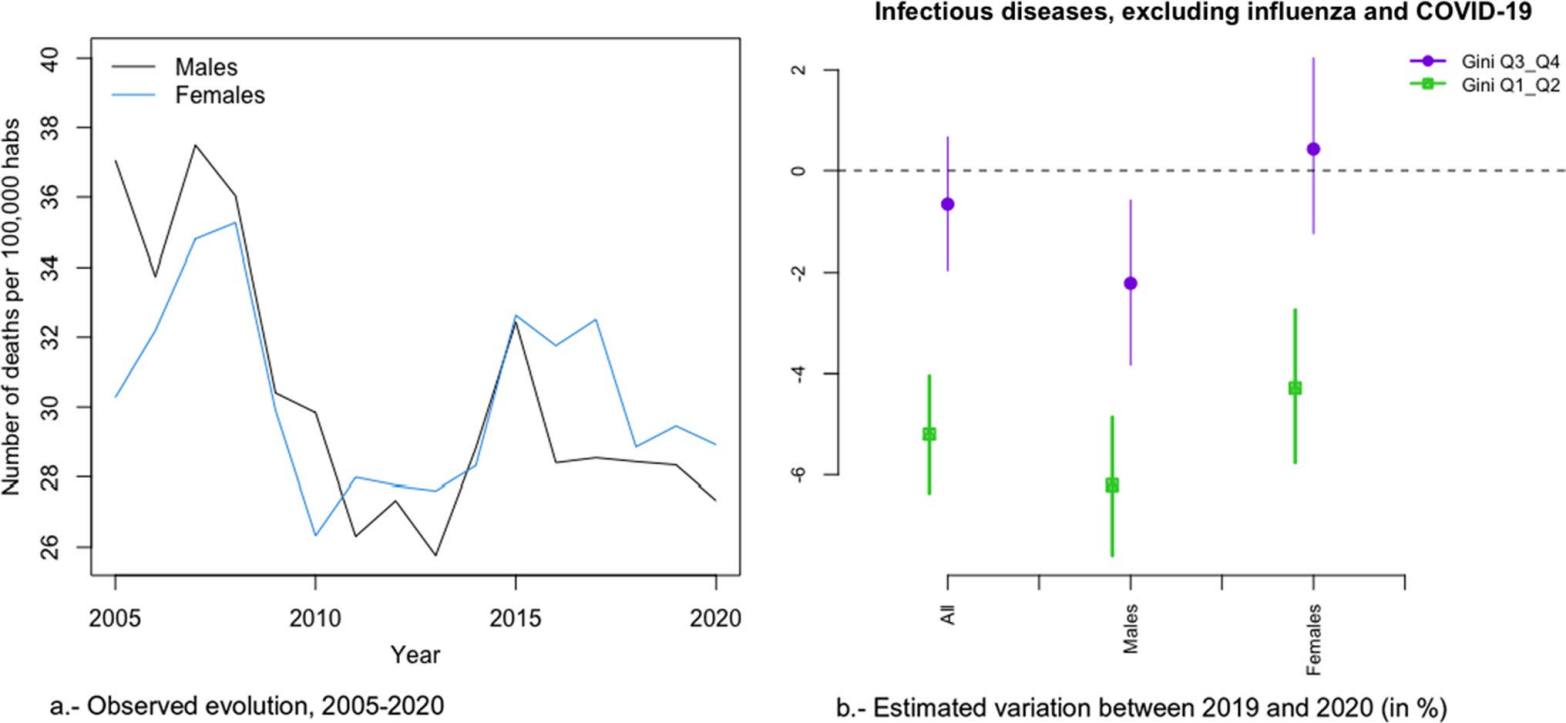
Evolution of mortality from infectious diseases, excluding influenza and COVID-19

**Fig. 11:**
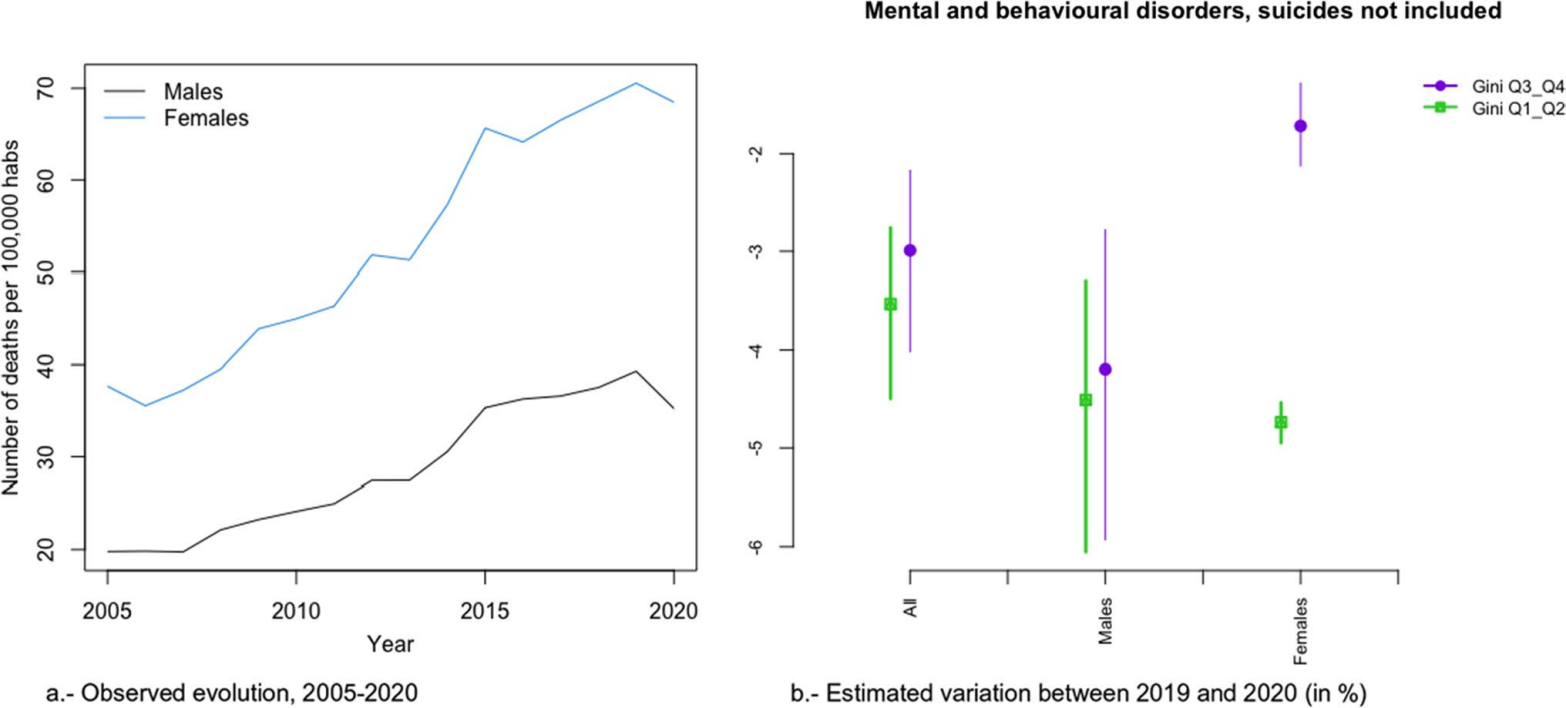
Evolution of mortality from mental and behavioural disorders, suicides not included

**Fig. 12:**
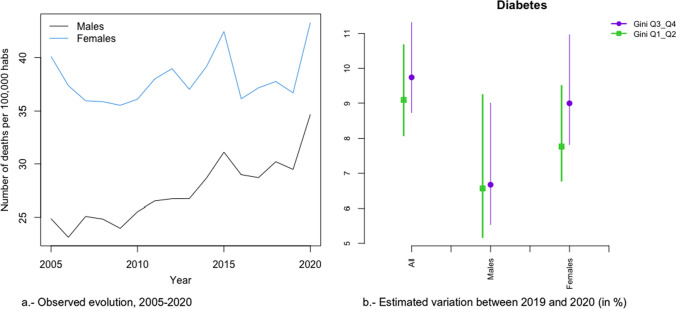
Evolution of mortality from diabetes

**Fig. 13:**
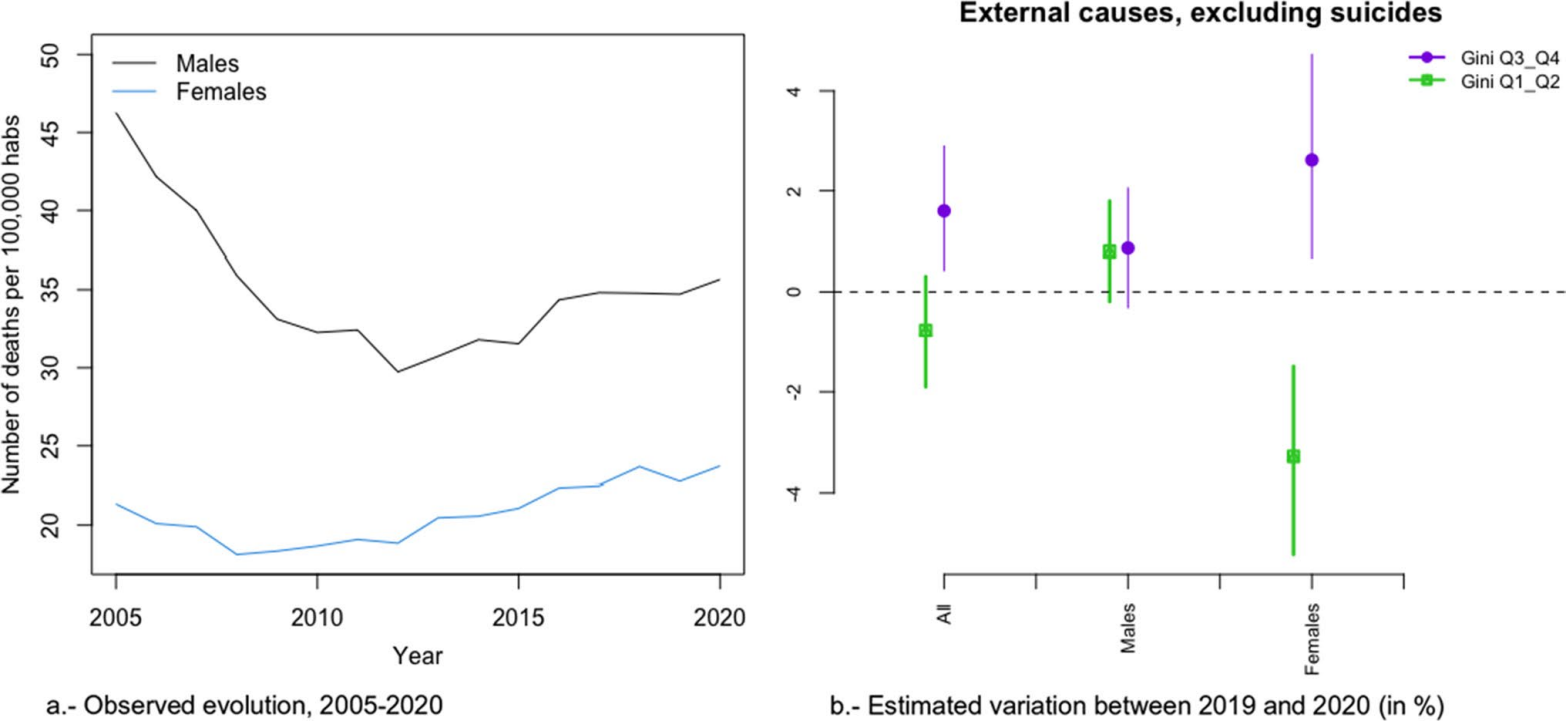
Evolution of mortality from external causes, excluding suicides

**Fig. 14:**
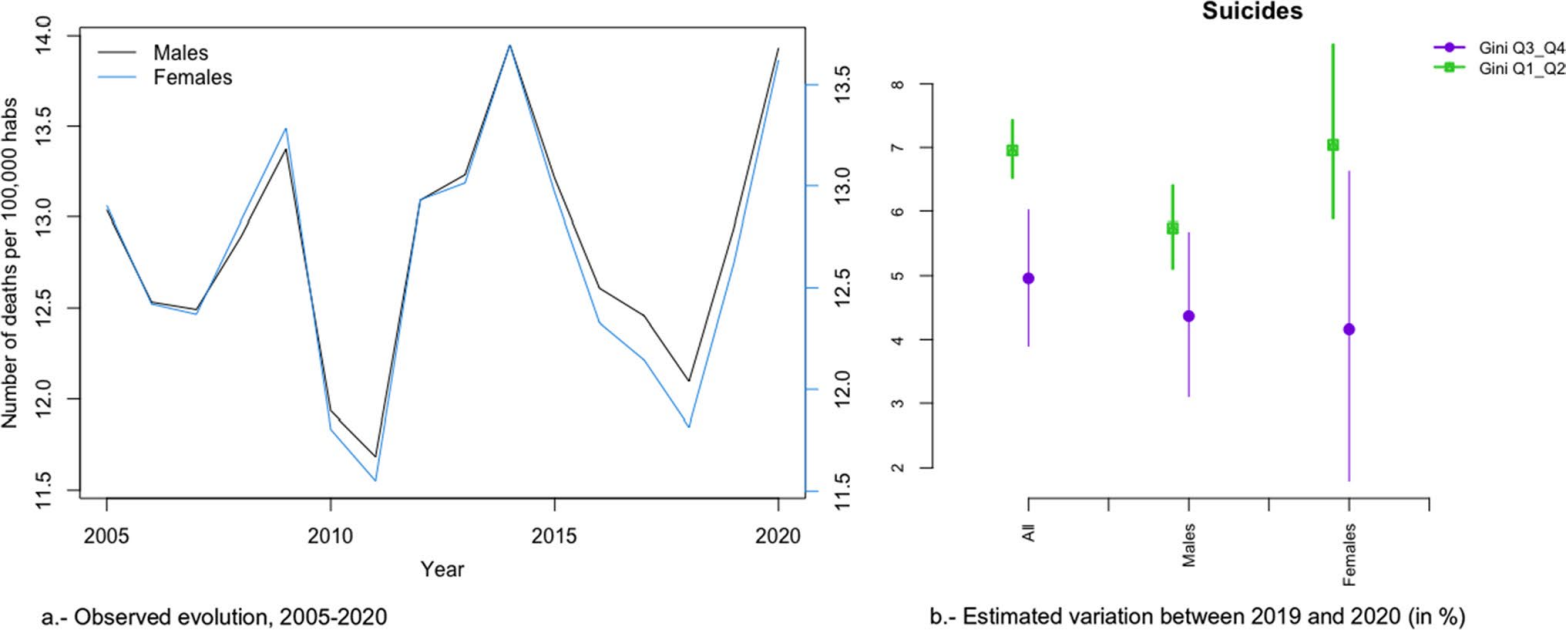
Evolution of mortality from suicides

**Fig. 15:**
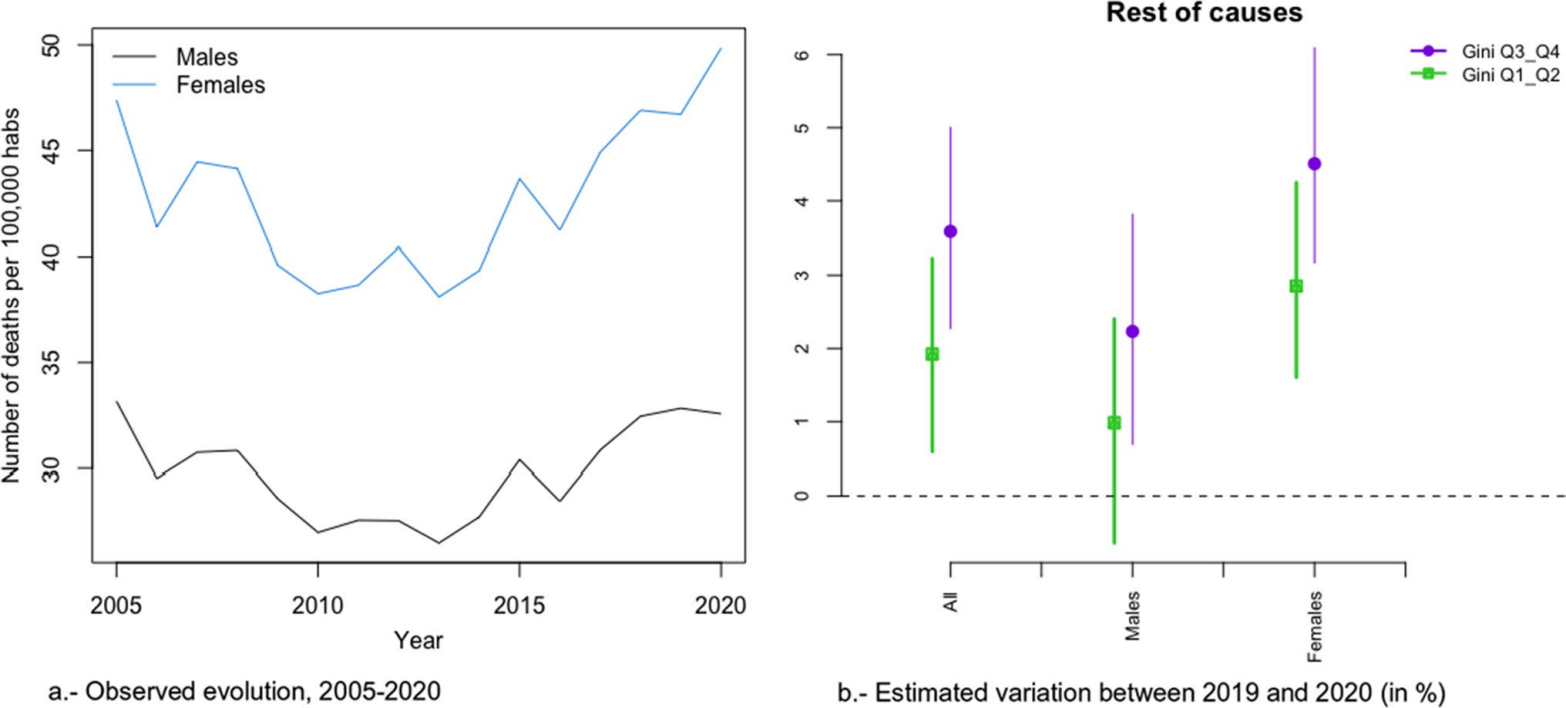
Evolution of mortality from rest of causes

**Table 3:** Results of the estimation of the models

All-cause mortality	All		Men		Women	
Average net income per person less than €10,912 [Q3, Q4]	1.045(0.997, 1.096)		1.053(1.004,1.105)		1.037(0.988,1.088)	
Trend [2005–2017]	Gini < 34.22	Gini ≥ 34.22	Gini < 34.22	Gini ≥ 34.22	Gini < 34.22	Gini ≥ 34.22
2018	1.058(1.046,1.070)	1.060(1.048,1.073)	1.049(1.037,1.062)	1.048(1.035,1.061)	1.067(1.055,1.080)	1.076(1.062,1.089)
2019	1.027(1.015,1.039)	1.044(1.031, 1.056)	1.026(1.014,1.039)	1.035(1.022,1.047)	1.030(1.018,1.043)	1.055(1.042,1.068)
2020	1.178(1.163,1.193)	1.229(1.213, 1.246)	1.170(1.155,1.186)	1.204(1.188,1.222)	1.186(1.170,1.202)	1.245(1.228,1.263)
Model	Negative binomial		Inflated		Inflated	
All-cause mortality excluding COVID-19						
Average net income per person less than €10,912 [Q3, Q4]	1.055(1.009,1.103)		1.065(1.019,1.112)		1.045(0.996,1.096)	
Trend [2005–2017]	Gini < 34.22	Gini ≥ 34.22	Gini < 34.22	Gini ≥ 34.22	Gini < 34.22	Gini ≥ 34.22
2018	1.069(1.060,1.078)	1.073(1.064,1.082)	1.061(1.052,1.070)	1.060(1.051,1.070)	1.077(1.068,1.087)	1.087(1.077,1.097)
2019	1.040(1.032,1.050)	1.047(1.038,1.055)	1.039(1.030,1.048)	1.036(1.027,1.046)	1.043(1.034,1.053)	1.058(1.048,1.068)
2020	1.037(1.028,1.047)	1.045(1.035,1.055)	1.027(1.017,1.037)	1.023(1.013,1.034)	1.048(1.037,1.059)	1.068(1.056,1.080)
Model	Negative binomial		Negative binomial		Negative binomial	

Relative risks (95% credible intervals)

Reference categories in brackets. Q3 and Q4 for average net income per person and 2005–2017 for the trend. Models adjusted by Gini index, percentage of population aged 65 or over, individual heterogeneity (at province level), time trend and population (as offset)

**Table 4:** Results of the estimation of the models. Relative risks (95% credible intervals)

Cardiovascular diseases	All		Men		Women	
Average net income per person less than €10,912 [Q3, Q4]	1.107(1.028,1.192)		1.104(1.028,1.184)		1.111(1.025,1.203)	
Trend [2005–2017]	Gini < 34.22	Gini ≥ 34.22	Gini < 34.22	Gini ≥ 34.22	Gini < 34.22	Gini ≥ 34.22
2018	0.992(0.986,0.998)	0.999(0.991,1.007)	1.005(0.997,1.014)	1.012(1.001,1.023)	0.979(0.971,0.987)	0.989(0.978,0.999)
2019	0.952(0.946,0.958)	0.970(0.962,0.979)	0.978(0.969,0.986)	0.988(0.977,1.001)	0.932(0.925,0.940)	0.952(0.942,0.962)
2020	0.968(0.962,0.975)	0.980(0.971,0.989)	0.993(0.984,1.002)	0.991(0.978,1.004)	0.946(0.938,0.954)	0.972(0.960,0.984)
Model	Inflated		Inflated		Inflated	
Cancer						
Average net income per person less than €10,912 [Q3, Q4]	0.991(0.939,1.047)		1.004(0.946,1.067)		0.972(0.924,1.021)	
Trend [2005–2017]	Gini < 34.22	Gini ≥ 34.22	Gini < 34.22	Gini ≥ 34.22	Gini < 34.22	Gini ≥ 34.22
2018	1.035(1.030,1.041)	1.036(1.029,1.043)	1.029(1.023,1.036)	1.027(1.019,1.035)	1.051(1.043,1.058)	1.055(1.046,1.065)
2019	1.032(1.026,1.037)	1.029(1.022,1.036)	1.019(1.013,1.026)	1.013(1.005,1.021)	1.054(1.047,1.061)	1.058(1.048,1.067)
2020	1.022(1.016,1.029)	1.020(1.010,1.029)	1.004(0.995,1.011)	0.995(0.984,1.006)	1.056(1.046,1.065)	1.062(1.048,1.076)
Model	Inflated		Inflated		Inflated	
Respiratory diseases excluding influenza and COVID-19						
Average net income per person less than €10,912 [Q3, Q4]	1.103(1.024,1.188)		1.127(1.048,1.212)		1.072(0.986,1.166)	
Trend [2005–2017]	Gini < 34.22	Gini ≥ 34.22	Gini < 34.22	Gini ≥ 34.22	Gini < 34.22	Gini ≥ 34.22
2018	1.148(1.138,1.159)	1.159(1.144,1.173)	1.117(1.103,1.132)	1.104(1.085,1.122)	1.191(1.174,1.207)	1.232(1.211,1.255)
2019	1.023(1.0129,1.033)	1.018(1.005,1.031)	1.009(0.995,1.022)	0.985(0.967,1.002)	1.044(1.029,1.060)	1.065(1.045,1.085)
2020	0.917(0.907,0.927)	0.901(0.887,0.916)	0.915(0.902,0.928)	0.893(0.875,0.912)	0.921(0.906,0.937)	0.915(0.894,0.937)
Model	Inflated		Inflated		Inflated	

Reference categories in brackets. Q3 and Q4 for average net income per person and 2005–2017 for the trend. Models adjusted by Gini index, percentage of population aged 65 or over, individual heterogeneity (at province level), time trend and population (as offset)

**Table 5:** Results of the estimation of the models. Relative risks (95% credible intervals)

Influenza	All		Men		Women	
Average net income per person less than €10,912 [Q3, Q4]	0.840(0.695,1.013)		0.847(0.678,1.053)		0.855(0.687,1.062)	
Trend [2005–2017]	Gini < 34.22	Gini ≥ 34.22	Gini < 34.22	Gini ≥ 34.22	Gini < 34.22	Gini ≥ 34.22
2018	6.065(5.259,7.016)	5.200(4.423,6.078)	5.441(4.871,6.091)	4.725(4.001,5.535)	6.216(5.656,6.839)	4.415(3.785,5.124)
2019	4.800(4.142,5.564)	4.363(3.733,5.097)	4.339(3.877,4.861)	4.252(3.636,4.978)	4.439(4.019,4.903)	3.746(3.245,4.325)
2020	3.058(2.573,3.631)	2.991(2.484,3.609)	2.717(2.381,3.100)	2.984(2.477,3.602)	2.365(2.087,2.678)	2.453(2.057,2.930)
Model	Negative binomial		Inflated		Inflated	
Alzheimer						
Average net income per person less than €10,912 [Q3, Q4]	0.931(0.857,1.011)		0.953(0.880,1.031)		0.919(0.842,1.003)	
Trend [2005–2017]	Gini < 34.22	Gini ≥ 34.22	Gini < 34.22	Gini ≥ 34.22	Gini < 34.22	Gini ≥ 34.22
2018	1.232(1.217,1.247)	1.206(1.188,1.226)	1.254(1.233,1.276)	1.211(1.185,1.238)	1.214(1.195,1.233)	1.198(1.176,1.223)
2019	1.222(1.206,1.237)	1.186(1.167,1.206)	1.257(1.234,1.278)	1.201(1.173,1.228)	1.202(1.183,1.221)	1.178(1.155,1.202)
2020	1.280(1.263,1.297)	1.222(1.198,1.247)	1.280(1.255,1.307)	1.206(1.171,1.242)	1.275(1.254,1.296)	1.233(1.202,1.263)
Model	Inflated		Inflated		Inflated	
Digestive diseases						
Average net income per person less than €10,912 [Q3, Q4]	1.084(1.0113,1.162)		1.107(1.028,1.190)		1.062(0.987,1.143)	
Trend [2005–2017]	Gini < 34.22	Gini ≥ 34.22	Gini < 34.22	Gini ≥ 34.22	Gini < 34.22	Gini ≥ 34.22
2018	1.069(1.056,1.083)	1.082(1.066,1.099)	1.055(1.039,1.072)	1.052(1.032,1.073)	1.077(1.058,1.097)	1.105(1.082,1.130)
2019	1.084(1.070,1.097)	1.091(1.075,1.108)	1.070(1.053,1.089)	1.061(1.039,1.084)	1.090(1.073,1.109)	1.116(1.094,1.140)
2020	1.063(1.047,1.078)	1.066(1.045,1.087)	1.053(1.032,1.074)	1.045(1.017,1.074)	1.082(1.060,1.105)	1.100(1.068,1.132)
Model	Inflated		Inflated		Inflated	

Reference categories in brackets. Q3 and Q4 for average net income per person and 2005–2017 for the trend. Models adjusted by Gini index, percentage of population aged 65 or over, individual heterogeneity (at province level), time trend and population (as offset)

**Table 6:** Results of the estimation of the models. Relative risks (95% credible intervals)

Infectious diseases excluding COVID-19 and influenza	All		Men		Women	
Average net income per person less than €10,912 [Q3, Q4]	1.023(0.932,1.123)		1.033(0.938,1.138)		1.010(0.919,1.112)	
Trend [2005–2017]	Gini < 34.22	Gini ≥ 34.22	Gini < 34.22	Gini ≥ 34.22	Gini < 34.22	Gini ≥ 34.22
2018	0.996(0.956,1.037)	0.949(0.901,0.989)	0.976(0.934,1.020)	0.939(0.897,0.983)	1.022(0.973,1.071)	0.963(0.915,1.013)
2019	0.948(0.909,0.989)	0.945(0.905,0.987)	0.932(0.891,0.976)	0.934(0.890,0.981)	0.965(0.918,1.013)	0.951(0.903,1.001)
2020	0.899(0.851,0.949)	0.939(0.887,0.993)	0.875(0.824,0.928)	0.913(0.856,0.975)	0.924(0.865,0.985)	0.955(0.892,1.024)
Mental and behavioural disorders excluding suicides						
Average net income per person less than €10,912 [Q3, Q4]	0.893(0.769,1.036)		0.928(0.802,1.073)		0.878(0.754,1.023)	
Trend [2005–2017]	Gini < 34.22	Gini ≥ 34.22	Gini < 34.22	Gini ≥ 34.22	Gini < 34.22	Gini ≥ 34.22
2018	1.296(1.257,1.338)	1.366(1.323,1.410)	1.305(1.261,1.353)	1.384(1.335,1.436)	1.285(1.263,1.306)	1.359(1.332,1.387)
2019	1.311(1.269,1.358)	1.389(1.343,1.439)	1.307(1.259,1.361)	1.390(1.337,1.449)	1.286(1.264,1.307)	1.397(1.369,1.425)
2020	1.265(1.212,1.321)	1.347(1.289,1.407)	1.248(1.183,1.316)	1.332(1.258,1.409)	1.225(1.202,1.248)	1.373(1.340,1.407)
Model	Negative binomial		Negative binomial		Inflated	
Diabetes mellitus						
Average net income per person less than €10,912 [Q3, Q4]	1.105(0.958,1.273)		1.068(0.920,1.240)		1.130(0.981,1.303)	
Trend [2005–2017]	Gini < 34.22	Gini ≥ 34.22	Gini < 34.22	Gini ≥ 34.22	Gini < 34.22	Gini ≥ 34.22
2018	1.020(0.982,1.059)	1.053(1.012,1.093)	1.089(1.043,1.130)	1.127(1.078,1.172)	0.981(0.941,1.020)	1.014(0.971,1.056)
2019	1.049(1.003,1.093)	1.087(1.039,1.134)	1.131(1.083,1.176)	1.172(1.117,1.221)	0.999(0.952,1.042)	1.044(0.994,1.091)
2020	1.144(1.084,1.209)	1.193(1.129,1.262)	1.205(1.139,1.285)	1.250(1.179,1.331)	1.076(1.016,1.141)	1.137(1.071,1.210)
Model	Negative binomial		Negative binomial		Negative binomial	

Reference categories in brackets. Q3 and Q4 for average net income per person and 2005–2017 for the trend. Models adjusted by Gini index, percentage of population aged 65 or over, individual heterogeneity (at province level), time trend and population (as offset)

**Table 7:** Results of the estimation of the models. Relative risks (95% credible intervals)

Urinary system diseases	All		Men		Women	
Average net income per person less than €10,912 [Q3, Q4]	1.115(1.0192,1.219)		1.090(0.997,1.191)		1.136(1.030,1.252)	
Trend [2005–2017]	Gini < 34.22	Gini ≥ 34.22	Gini < 34.22	Gini ≥ 34.22	Gini < 34.22	Gini ≥ 34.22
2018	1.174(1.155,1.193)	1.219(1.195,1.243)	1.138(1.115,1.163)	1.175(1.147,1.207)	1.194(1.170,1.220)	1.246(1.214,1.279)
2019	1.188(1.168,1.208)	1.256(1.230,1.282)	1.176(1.150,1.201)	1.234(1.201,1.267)	1.210(1.183,1.237)	1.287(1.253,1.323)
2020	1.302(1.279,1.325)	1.402(1.368,1.437)	1.241(1.210,1.273)	1.322(1.276,1.369)	1.347(1.316,1.378)	1.461(1.416,1.510)
Model	Inflated		Inflated		Inflated	
External causes excluding suicides						
Average net income per person less than €10,912 [Q3, Q4]	0.957(0.877,1.044)		1.006(0.924,1.095)		0.881(0.787,0.986)	
Trend [2005–2017]	Gini < 34.22	Gini ≥ 34.22	Gini < 34.22	Gini ≥ 34.22	Gini < 34.22	Gini ≥ 34.22
2018	1.023(0.998,1.051)	1.058(1.028,1.087)	0.985(0.961,1.010)	1.014(0.986,1.042)	1.100(1.062,1.143)	1.135(1.090,1.179)
2019	1.020(0.994,1.047)	1.071(1.042,1.101)	0.994(0.968,1.020)	1.023(0.993,1.053)	1.079(1.042,1.119)	1.158(1.114,1.204)
2020	1.012(0.975,1.050)	1.088(1.046,1.133)	1.002(0.967,1.038)	1.032(0.990,1.075)	1.044(0.987,1.102)	1.189(1.122,1.260)
Model	Negative binomial		Negative binomial		Negative binomial	
Suicides						
Average net income per person less than €10,912 [Q3, Q4]	1.170(1.034 1.323)		1.221(1.088,1.369)		1.051(0.892,1.237)	
Trend [2005–2017]	Gini < 34.22	Gini ≥ 34.22	Gini < 34.22	Gini ≥ 34.22	Gini < 34.22	Gini ≥ 34.22
2018	1.025(0.995,1.055)	0.984(0.950,1.019)	1.009(0.976,1.042)	0.973(0.934,1.012)	1.094(1.042,1.148)	1.036(0.976,1.100)
2019	1.056(1.025,1.087)	0.996(0.960,1.033)	1.048(1.015,1.083)	0.997(0.956,1.038)	1.109(1.053,1.166)	1.028(0.960,1.098)
2020	1.129(1.092,1.168)	1.045(0.997,1.095)	1.108(1.066,1.152)	1.040(0.986,1.097)	1.187(1.115,1.266)	1.070(0.977,1.171)
Model	inflated		inflated		inflated	

Reference categories in brackets. Q3 and Q4 for average net income per person and 2005–2017 for the trend. Models adjusted by Gini index, percentage of population aged 65 or over, individual heterogeneity (at province level), time trend and population (as offset)

**Table 8:** Results of the estimation of the models. Relative risks (95% credible intervals)

Rest of causes	All		Men		Women	
Average net income per person less than €10,912 [Q3, Q4]	1.096(0.982,1.224)		1.103(0.991,1.228)		1.093(0.967,1.234)	
Trend [2005–2017]	Gini < 34.22	Gini ≥ 34.22	Gini < 34.22	Gini ≥ 34.22	Gini < 34.22	Gini ≥ 34.22
2018	1.082(1.052,1.117)	1.077(1.046,1.112)	1.065(1.035,1.099)	1.071(1.039,1.107)	1.091(1.057,1.128)	1.081(1.046,1.118)
2019	1.100(1.066,1.135)	1.111(1.076,1.147)	1.082(1.049,1.117)	1.100(1.064,1.139)	1.110(1.071,1.149)	1.117(1.078,1.158)
2020	1.122(1.073,1.171)	1.151(1.101,1.204)	1.093(1.042,1.144)	1.125(1.071,1.182)	1.142(1.088,1.198)	1.168(1.112,1.228)
Model	Negative binomial		Negative binomial		Negative binomial	

Reference categories in brackets. Q3 and Q4 for average net income per person and 2005–2017 for the trend. Models adjusted by Gini index, percentage of population aged 65 or over, individual heterogeneity (at province level), time trend and population (as offset)

As can be seen in Fig. 2a, the crude mortality rate decreased from 2005 to 2010, increasing afterwards, especially from 2012, before starting to fall again in 2019. This decrease was interrupted by the pandemic (Fig. 4a).

We estimated that during the study period (2005–2020) the risk of dying from all causes was 5.5% higher in provinces with lower socioeconomic levels (the first two quartiles of the average net income per person) (Table 3). This risk was higher for men (6.5%) than for women (4.5%, although this was different from zero only at 90% confidence). Note also how the pandemic (i.e., in 2020) did not change these estimates. The credible intervals of the relative risks for the variable average net income per person when COVID-19 was included overlap with the intervals when this cause of mortality was excluded (in all cases and in both men and women) (Table 3). Based on these results, we were able to affirm that the COVID-19 pandemic did not modify the gap in the risk of dying between the less economically disadvantaged provinces and the more affluent ones.

However, this picture changes when we inspect the estimators of the interaction between the temporal evolution of mortality and inequality, measured through the Gini index (Table 3 and Figs. 2b and 3b). In those provinces with higher inequality (the last two quartiles of the Gini index), the risk of dying was 17.72% higher in 2020 than in 2019 (Relative risk considering 2019 as reference—RR from now on -: 1.772), while than in those with lower inequality (the first two quartiles), the risk was 14.70% higher (RR = 1.147) (Fig. 3b). Furthermore, these differences in risk between the provinces with the highest and lowest inequalities were statistically significant (the 95% credible intervals did not overlap, see the first row of Table 3 and Fig. 3b). Thus, although the COVID-19 pandemic increased the risk of dying in all Spanish provinces, the increase was more pronounced in those provinces with greater inequality.

### Trends in All Causes of Death by Gender

These differences occurred in both sexes. The risk of dying in 2020 compared to 2019, was 16.33% higher in the case of men (RR = 1.163) and 18.01% higher in the case of women (RR = 1.180) in the provinces with greater inequality, while in the provinces with less inequality, it was 14.03% in the case of men (RR = 1.140) and 15.15% in the case of women (RR = 1.152) (Fig. 3b). However, the differences in the increases in risk (between 2019 and 2020) between the most and least unequal provinces were 2.30% for men (RR = 1.023) and 2.83% (RR = 1.028) for women; i.e., practically equal. Therefore, it does not seem that the COVID-19 pandemic has led to a difference in the increased risk of dying by gender.

That said, things change when the indirect effects of the pandemic on mortality are considered (compare rows 1 and 2 of Table 3 and, in particular, Fig. 3b). In the case of women, the risk of dying (from causes other than COVID-19) changed its trend in 2020. Note, furthermore, that also in the case of women the increase in risk was greater in those provinces that were more unequal and that these increases were statistically different.

### Trends in Specific Causes of Death

As regards the specific causes, throughout the period considered (2005–2020) the risk of dying from cardiovascular diseases (both sexes), respiratory diseases (in the case of men), digestive diseases (in the case of men), suicide (in the case of men), and diseases of the urinary system (in the case of women) was higher in provinces with lower socioeconomic levels. Note also that these risks were practically double (in suicides four times more) than the risk of dying from all causes.

The risk of dying from external causes in the case of women, however, was lower in the provinces with lower socioeconomic levels (approximately 12% lower – RR = 0.88-). In the rest of the causes, there were no differences in the risk of dying according to the socioeconomic level of the provinces throughout the entire period considered.

To facilitate the interpretation of the effects of COVID-19 on the risk of dying from other causes, we will distinguish between those that presented a decreasing evolution since well before 2020, those that presented an increasing evolution, and those that presented an oscillating (increasing and decreasing) evolution.

The causes that presented a decreasing evolution since before 2020 were: cardiovascular diseases (Fig. 4a), cancer (Fig. 5a), respiratory diseases, excluding influenza and COVID-19 (Fig. 6a), influenza (Fig. 7a), and infectious diseases, also excluding influenza and COVID-19 (Fig. 10a).

Among these causes, there was a change in trend (with an increased risk of dying in 2020) in cardiovascular diseases (both sexes) and in cancer (only in women) (Figs. 4 and 5). Only in the case of cardiovascular diseases was the risk of dying different between the most unequal provinces (higher risk in women) and the less unequal (higher risk in men) (Figs. 4b and 5b). However, for both cardiovascular diseases and cancer there were differences by gender (greater increases in risk in 2020 in the case of women).

In the risk of dying from respiratory diseases and from infectious diseases (in both cases excluding COVID-19 and the influenza), as well as from the influenza, there was no change in trend, but the risk continued to decrease in 2020 (Figs. 6, 7 and 10). However, in the case of infectious diseases and influenza, the reduction in risk was greater in provinces with less inequality for both men and women (Fig. 7b and 10b). In contrast, in respiratory diseases, the reduction in risk was greater in provinces that were more unequal, albeit only for women (Fig. 6b). With the exception of infectious diseases (Fig. 10b), the differences (according to inequality in the provinces) in the variations in the risk of dying between 2019 and 2020 were greater in women (Figs. 6b and 7b).

Alzheimer’s (Fig. 8a), digestive system diseases (Fig. 9a), mental and behavioural disorders (Fig. 11a), diabetes (Fig. 12a), external causes (Fig. 13a) and the rest of the causes (Fig. 15a) presented an increasing evolution since before 2020.

Among these causes, changes in trend (from an increase before 2020 to a reduction in 2020) were estimated in the risks of dying from digestive system diseases (Fig. 10), mental and behavioural disorders (Fig. 11), and external causes (Fig. 13).

In the risk of dying from digestive system diseases, there were no differences due to inequality in the provinces or by gender (Fig. 9b). However, the reduction in the risk of dying from mental and behavioural disorders (Fig. 11b) and from external causes (Fig. 13b) was different according to the inequality in the provinces (greater risk reduction in the less unequal provinces) and in gender (only in women). Even in 2020, while the risk from external causes increased in the most unequal provinces, it decreased in the least unequal provinces (Fig. 13b).

Neither in the risk of dying from diabetes (Fig. 12b), nor for the rest of the causes (Fig. 15b) were any differences found due to inequality by provinces and by gender. In the risk of dying from Alzheimer’s (Fig. 8b), as in diabetes and other causes, while there was no change in the trend in the increased risk of dying in 2020, there were differences depending on the inequality of the provinces (greater increases in risk in less unequal provinces), although not by gender.

Mortality due to suicides (Fig. 14a) presented an oscillating evolution. In this case, there was an increase in the risk of dying in 2020, although this was not found to be different according to inequality by province (greater, although not statistically significant, in provinces with less inequality) or according to gender (Fig. 14b).

Finally, we found that the variations in the risk of dying between 2019 and 2020 according to inequality were greater in the case of COVID-19 than in the other causes. In this sense, while the difference in risk between the most unequal and the least unequal provinces was 3.09 percentage points in mortality from all causes (Relative risk, taking the least unequal provinces as reference: 1.039) (2.36 in the case of men—Relative risk: 1.0236- and 2.96 in the case of women—Relative risk: 1.0296-; Relative risk between sexes, taking men as the reference category: 1.006), mortality excluding COVID-19 decreased 0.18 percentage points (Relative risk: 1.0018) (increasing 0.15 in men—Relative risk: 1.0015- and decreasing 0.52 in women—Relative risk: 0.948; Relative risk between sexes, taking men as the reference category: 0.947).

Among the specific causes, we would like to highlight an increase in the differences between the most and the least unequal provinces in external (excluding suicide) causes (2.37 points, 0.06 in men and 5.91 in women), diseases of the urinary system (2.03 points—Relative risk: 1.023-, 1.58 in men—Relative risk: 1.0158- and 2.22 in women—Relative risk: 1.022; Relative risk between sexes, taking men as the reference category: 1.006), the rest of causes (1.66 points—Relative risk: 1.0166-, 1.25 men —Relative risk: 1.0125- and 1.65 womenRelative risk: 1.0165-; Relative risk between sexes, taking men as the reference category: 1.004) and in cardiovascular only in women (0.67 points—Relative risk: 1.0067-). On the contrary, in suicides (2.00 points Relative risk: 1.020-, 1.38 men—Relative risk: 1.0138- and 2.88 women—Relative risk: 1.0288-; Relative risk between sexes, taking men as the reference category: 1.015), Alzheimer (1.67 points—Relative risk: 1.0167-, 1.41 men—Relative risk: 1.0141- and 1.42 women -Relative risk: 1.0142; Relative risk between sexes, taking men as the reference category: 1.000) and cardiovascular, only men (1.23 points—Relative risk: 1-0123-), the increase in risk was greater in the less unequal provinces. Note that, with the exception of Alzheimer’s, the variations in risk were greater in women. In the other causes, either there was no difference in risk between 2019 and 2020 (diabetes) or that risk was reduced.

## Discussion

Our main finding was that the increased risk of dying in 2020 because of the COVID-19 pandemic was greater in the Spanish provinces with greater inequality, at least when assessed by the Gini index. Note that, we found that inequality, measured by the Gini index, and not so much income level, was the variable that best reflected the impact COVID-19 had on the risk of dying.

In line with our results, de Souza et al*.* point out that the social inequality of municipalities in the state of São Paulo (Brazil) and measured by the Gini index, was the variable with the greatest weight (together with density) in explaining the COVID-19 mortality [24]. Eichenbaum and Tate, using the Index of Concentration at the Extremes, a metric which captures socio-spatial and economic polarization, found that the counties of Georgia, USA, with the largest income disparity had 1.7 times the case-mortality rate compared to the most privileged counties [22].

Here, we have also provided evidence to answer the questions raised earlier.

First, the pandemic has exacerbated socioeconomic inequalities in mortality. Although it is true that at the end of the first semester of the pandemic Marmot and Allen suggested that COVID-19 would amplify inequalities [43], very few studies have confirmed this. Among them, we will mention Simon et al*.*, who found that the long-standing mortality advantage in the Latino population relative to the White population in Los Angeles County was reversed in 2020 [9], and Kontopantelis et al*.* who pointed out that inequalities between socioeconomic and geographic groups resulting from the COVID-19 pandemic are more pronounced than previously reported [11]. In fact, these inequalities in the outcomes of COVID-19 increased as the pandemic progressed. Thus, Bacigalupe et al*.* show that, in Spain, studies based on socioeconomic data of the census revealed an increase in inequalities, especially in women, between the first and second waves (July–December 2020) [5]. Griffith et al*.* point out that, contrary to previous months, the strong spatial patterning during autumn 2020 in England and Wales was almost entirely explained by deprivation. Furthermore, as mortality declines it does not do so equally, evolving more slowly in more deprived areas [26].

Second, although not directly, COVID-19 led to gender differences in the variations in the risk of dying between 2019 and 2020; higher in the case of women. In this sense, the risk of dying from causes other than COVID-19 changed its trend in 2020 only in the case of women. Furthermore, this increase in risk was greater in those provinces that were more unequal. Thus, the pandemic, in terms of its indirect effects, would have amplified gender inequalities in mortality. In fact, in a non-systematic review of data, Flor et al*.* find that there have been intensified levels of pre-existing non health-related inequalities between women and men during the COVID-19 pandemic [44]. Our findings are in line with those found in other studies [2, 5, 45], especially at the ecological level, because at the individual level either no differences were found by gender in the social gradient of the effects of COVID-19 on mortality [46] or when found, were higher in men than in women [17, 20].

Third, as regards the specific causes of mortality, there was a change in trend, from a decreasing or from an oscillating evolution since before 2020, to a growth in 2020 in mortality from cardiovascular diseases, suicide, and cancer, albeit the latter only in women. Among the causes that were already growing before 2020, diabetes (both sexes) and Alzheimer’s (only in women) grew at a faster rate. However, only in cardiovascular diseases and Alzheimer’s did the increased risk of dying differ between the most and the least unequal provinces. The increase in the risk of dying was different by gender (greater in women) in cardiovascular diseases and in cancer.

Our results are partly consistent with other studies. Konstantinoudis et al. find an effect of the pandemic on mortality due to myocardial infarction [18]. Simon et al*.* found that Latino individuals experienced an increase in heart disease annual age-adjusted mortality rates from 2019 to 2020 (although also in diabetes) that was not observed among White individuals [9]. The excess in years of life lost to cardiovascular diseases (and also diabetes) in the year 2020 was also found by Kontopantelis et al*.* [11]. However, they too do not find significant pandemic-related changes in year of list lost due to cancer and other indirect deaths (including drug-related, alcohol-specific, suicides, fatal accidents, and all other causes) [11]. Unlike us, Orellana and de Souza, find that despite the overall decrease in suicides in Brazil over the period assessed, substantial excess suicides were observed in regions that are historically more prone to health and socioeconomic inequalities [47].

Last, we found that the variations in the risk of dying from all causes between 2019 and 2020 according to inequality were greater than the risk of dying when COVID-19 is excluded. We also estimate differences in the increases in the risk of dying from specific causes according to the inequality of the provinces, albeit lower than in the case of COVID-19. These differences were greater in the provinces with more inequality in some of the causes (external causes—excluding suicides -, diseases of the urinary system, the rest of causes and in cardiovascular, in this case only women) and minor in others (suicide, Alzheimer’s and cardiovascular, only in men). However, except for the risk of dying from Alzheimer’s, we found that variations were consistently greater for women.

Our results differ in part from those provided by the Nuffield Trust [8], according to whom the rates of death in the most deprived areas are twice those of the most affluent for both COVID-19 and other causes. However, it must be said that these results refer to England and the first wave, so they could not be completely generalized. In addition, the results were not adjusted for possible confounders.

Our study may have some limitations. The most important being that we used an ecological design, thus leading to the possibility of an ecological fallacy. Therefore, it should be noted that, when interpreting the results, no inferences should be made at the individual level. It should also be noted, however, that several cohort studies [28–30] point out that there were inequalities in the outcomes of COVID-19, mortality in particular, both at the individual and the community contextual levels. Furthermore, ecological designs imply the existence of unmeasured confounding bias. That said, we have attempted to control for this bias in terms of both observed and unobserved confounding.

Futhermore, in using data at the provincial level, we could have incurred the ‘modifiable areal unit problem’ (MAUP) [48]. The MAUP, which refers to data aggregation in units for analysis, is a potential source of bias that affects spatial studies using aggregated data. Unfortunately, this problem is unsolvable since data are not available in Spain at a more disaggregated geographical level.

There could have been measurement errors in both the response variables and the explanatory variables. Regarding mortality from COVID-19, the COVID-19 Excess Mortality Collaborators estimated that, in the case of Spain, the ratio between excess mortality rate and reported COVID-19 mortality rate was 1.64 (1.59–1.68) [10]. The differences could be attributed to changes in the criteria for defining a death from COVID-19 [48], to underdiagnosis due to insufficient testing, or to other pandemic-related effects related to a reduction in access to health care [10]. Thus, for example, until May 21, 2020 the Spanish Government considered a death by COVID-19 to be someone who had presented a positive PCR result and thereafter is both the one who had a positive result on some test (PCR or fast test) and the one who presented symptoms at some point and a sanitary professional classified them as a possible case, but they did not have a diagnostic test with a positive result [49].

As regards the explanatory variables, the INE estimates both the average income per person and the Gini index from the information provided in the income tax returns [32]. However, in Spain people who have earned less than €22,000 per annum are not required to file a tax return. Thus, the INE does not have information on the most economically disadvantaged people. Nevertheless, given that the presence of measurement errors tends to underestimate the effect of the variable measured with error [50], it is very likely that our findings are indicative of greater inequality and its effects on the risks of dying.

Finally, in some of the cases there were no deaths in some of the provinces. This could imply that the appropriate link for GLMMs is not a Poisson one, even though it is a counting variable. For this reason, we allowed the GLMM link to be bien negative binomial bien zero inflated Poisson. Furthermore, this excess of zeros in the outcome variable could have implied a reduction in statistical power. Since the sample size cannot be increased, we were able to increase the power, increasing the risk (i.e., probability of making a type I error), say from 5 to 10% for example, thus reducing the probability of making a type II error and therefore increased statistical power.

## Conclusion

As main conclusions we would point out: (i) the increased risk of dying in 2020 because of the COVID-19 pandemic was greater in the Spanish provinces with greater inequality; (ii) the pandemic has exacerbated socioeconomic inequalities in mortality and; (iii) although not directly, COVID-19 led to gender differences in the variations in the risk of dying between 2019 and 2020; higher in the case of women.

We believe that our results can be used by health authorities to know where and in which population groups future pandemics will have the greatest impact and, therefore, be able to take the appropriate measures to prevent their effects.

## Supplementary Information

Below is the link to the electronic supplementary material.

Supplementary file1 (DOCX 1268 KB)

## Data Availability

We used open data with free access. Statistics of deaths according to cause of death [in Spanish] [Available at: https://www.ine.es/dyngs/INEbase/es/operacion.htm?c=Estadistica_C&cid=1254736176780&menu=resultados&idp=1254735573175, last accessed on July 5, 2022]. Household Income Distribution Atlas [Available at: https://www.ine.es/en/experimental/atlas/exp_atlas_tab_en.htm, last accessed on July 5, 2022]. Continous Register Statistics. [Available at: https://www.ine.es/dyngs/INEbase/en/operacion.htm?c=Estadistica_C&cid=1254736177012&menu=resultados&secc=1254736195461&idp=1254734710990#!tabs-1254736195557, last accessed on July 5, 2022].

## Abbreviations

COVID-19: Coronavirus disease 2019
GLMM: Generalized linear mixed models
ICD-10: International Classification of Diseases, 10th edition
INE: Spanish National Institute of Statistics
INLA: Integrated nested Laplace approximation
MAUP: Modifiable areal unit problem
ONS: British Office for National Statistics
PC priors: Priors that penalize complexity
RR: Relative risk
WAIC: Watanabe-Akaike information criterion

## Code Availability

Code will be available at www.researchprojects.es.
