# Supplementary material for: Impact of the COVID-19 Pandemic on the Socioeconomic Inequalities in Mortality in Spanish Provinces
Source: J Epidemiol Glob Health. 2023 Jun 9;13(3):453–75. doi: 10.1007/s44197-023-00125-0 (PMC10250865; doi:10.1007/s44197-023-00125-0)

**Statistical methods**

For each outcome variable we chose the link in which the Bayesian model selection method of Watanabe-Akaike information criterion (WAIC)^[S1]^ of the fitted model was lower.

In detail, conditional to the true risk in the province $i$ on year $t$, the cases of the response variable ($Y_{it}$) occurring in each of the provinces on each year was distributed as a negative binomial or as a zero inflated Poisson.

$$Y_{it}\left| \theta_{it} \right.\sim Negative binomial\left( \theta_{it}{Population}_{it} \right)$$

$$Y_{it}\left| \theta_{it} \right.\sim Zero inflated Poisson\left( \theta_{it}{Population}_{it} \right)$$

where $\theta_{it}$ denoted $E\left( Y_{it} \right)=\theta_{it}$; $i=1,\ldots, 54$; $t$=2005, 2006,…, 2020; and ${Population}_{it}$ was the population at risk of being a case (death) in the province $i$ and on year $t$.

The link functions of the GLMMs were as follows:

$$\log\left( \theta_{it} \right)=\beta_{0}+\beta_{1} {income\_Q12}_{i}+\beta_{2} {Gini\_Q34}_{i}+\sum_{k=2}^{4} \beta_{3k} {Perc\_pop\_65\_or\_moreQ}_{ik} + \eta_{i}+\tau_{t}{Gini\_Q34}_{t} +offset(\log\left( {Population}_{it} \right))$$

where the subindexes $i$ and $t$ indicated the province, and the year, respectively; *income_Q12_i_* denoted whether the province is located in one of the first two quartiles of the average income per person; *Gini_Q34_i_* denoted whether the province is located in one of the last two quartiles of the Gini index*; Perc_pop_65_or_moreQ_ik_* the percentage of population aged 65 and over in 2020 (in quartiles, taking the first quartile as the reference category): $\eta_{i}, \tau_{t}$ denoted random effects; and $\beta s$ were the coefficients of the explanatory and control variables ($e^{\beta}$ was the relative risk associated with each of them).

We included two random effects in the models. First, $\eta_{i}$, a random effect indexed on the province. This random effect was unstructured (independent and identically distributed random effects, iid), and captured individual heterogeneity, that is to say, unobserved confounders specific to the province and invariant in time.

Second, we included $\tau_{t}$, a structured random effect (random walk of order one, rw1) indexed on time. Following the integrated nested Laplace approximations (INLA) approach^[S2,S3]^ when, as in our case, the random effects are indexed on a continuous variable, they can be used as smoothers to model non-linear dependency on covariates in the linear predictor. With this random effect we captured the temporal dependency, that is, the trend, which we allowed to be non-linear.

Note that we included in the models this random effect interacting with Gini_Q34. In fact, for each cause we were interested in evaluating the mortality trend (possibly non-linear) distinguishing between the provinces located in the last two quartiles of the index of Gini (those with the greatest inequality) and in the first two quartiles (those with the least inequality) of the index.

Following the INLA approach, random effects were defined using a multivariate Gaussian distribution with a zero mean and precision matrix kΣ, where *k* was a constant and Σ was a matrix that defined the dependence structure of the random effects^[S2,S3]^. In unstructured random effects (iid) Σ was a diagonal matrix of 1s, and in random walk random effects Σ was defined assuming that increments (in rw1, $\Delta u_{i}=u_{t}-u_{t-1}$) followed a Gaussian distribution with zero mean and a constant precision *k*^[S4,S5]^.

**References of the supplementary material**

S1.- Watanabe S. A widely applicable Bayesian information criterion. *Journal of Machine Learning Research* 2013; 14:867-897.

S2.- Rue H, Martino S, Chopin N. Approximate Bayesian inference for latent Gaussian models using integrated nested Laplace approximations (with discussion). *J R Stat Soc Series B Stat Methodol*. 2009; 71:319-392. doi:[j.1467-9868.2008.00700.x](file:///Users/marcsaez/Documentos/Treballs%20varis/COVID-19/Defunciones%20por%20sexo%20y%20edad%20(lista%20reducida)/j.1467-9868.2008.00700.x).

S3.- Rue H, Riebler A, Sørbye H, Illian JB, Simpson DP, Lindgren FK. 2017. Bayesian computing with INLA: A review. *Annual Reviews of Statistics and its Applications*, 4(March), 395-421. doi: [annurev-statistics-060116-054045](file:///Users/marcsaez/Documentos/Treballs%20varis/COVID-19/Defunciones%20por%20sexo%20y%20edad%20(lista%20reducida)/annurev-statistics-060116-054045).

S4. - Gómez-Rubio V. 2020. *Bayesian Inference with INLA.* London, United Kingdom: Chapman and Hall/CRC, Chapter 3.

S5.- R INLA project, 2022 [Available at: <http://www.r-inla.org/home>, last accessed on May 28, 2022].

**Table S1.- Mortality rates from specific causes, standardized by sex and age (per 100,000 inhabitants). Spain, autonomous communities and cities. Without stratifying and stratifying by gender, 2018.**

**Figure S1.- Scatter graph of the Gini index against the average net income per person, Spanish provinces, average 2015-2018.**


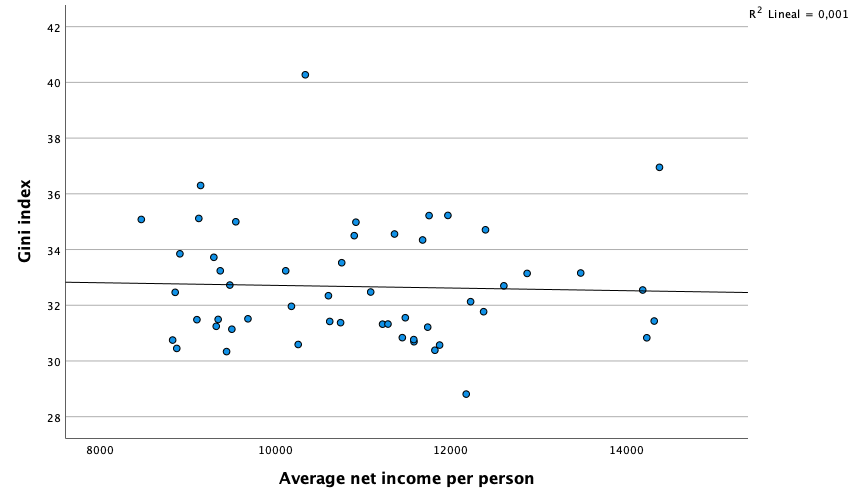

Supplement: Supplementary file 1 — Supplementary file1 (DOCX 1268 KB) [file 44197_2023_125_MOESM1_ESM.docx]
